# Comparative Genomic Analysis of the Class *Epsilonproteobacteria* and Proposed Reclassification to Epsilonbacteraeota (phyl. nov.)

**DOI:** 10.3389/fmicb.2017.00682

**Published:** 2017-04-24

**Authors:** David W. Waite, Inka Vanwonterghem, Christian Rinke, Donovan H. Parks, Ying Zhang, Ken Takai, Stefan M. Sievert, Jörg Simon, Barbara J. Campbell, Thomas E. Hanson, Tanja Woyke, Martin G. Klotz, Philip Hugenholtz

**Affiliations:** ^1^Australian Centre for Ecogenomics, School of Chemistry and Molecular Biosciences, The University of Queensland, St LuciaQLD, Australia; ^2^Department of Cell and Molecular Biology, College of the Environment and Life Sciences, University of Rhode Island, KingstonRI, USA; ^3^Department of Subsurface Geobiological Analysis and Research, Japan Agency for Marine-Earth Science and TechnologyYokosuka, Japan; ^4^Department of Biology, Woods Hole Oceanographic Institution, Woods HoleMA, USA; ^5^Microbial Energy Conversion and Biotechnology, Department of Biology, Technische Universität DarmstadtDarmstadt, Germany; ^6^Department of Biological Sciences, Life Science Facility, Clemson University, ClemsonSC, USA; ^7^School of Marine Science and Policy, College of Earth, Ocean, and Environment, Delaware Biotechnology Institute, University of Delaware, NewarkDE, USA; ^8^Department of Energy, Joint Genome Institute, Walnut CreekCA, USA; ^9^Department of Biology and School of Earth and Environmental Sciences, Queens College of the City University of New York, New YorkNY, USA; ^10^State Key Laboratory of Marine Environmental Science, Institute of Marine Microbes and Ecospheres, College of Ocean and Earth Sciences, Xiamen UniversityXiamen, China

**Keywords:** *Epsilonproteobacteria*, taxonomy, classification, genome, phylogenomics, Epsilonbacteraeota, evolution

## Abstract

The *Epsilonproteobacteria* is the fifth validly described class of the phylum Proteobacteria, known primarily for clinical relevance and for chemolithotrophy in various terrestrial and marine environments, including deep-sea hydrothermal vents. As 16S rRNA gene repositories have expanded and protein marker analysis become more common, the phylogenetic placement of this class has become less certain. A number of recent analyses of the bacterial tree of life using both 16S rRNA and concatenated marker gene analyses have failed to recover the *Epsilonproteobacteria* as monophyletic with all other classes of Proteobacteria. In order to address this issue, we investigated the phylogenetic placement of this class in the bacterial domain using 16S and 23S rRNA genes, as well as 120 single-copy marker proteins. Single- and concatenated-marker trees were created using a data set of 4,170 bacterial representatives, including 98 *Epsilonproteobacteria*. Phylogenies were inferred under a variety of tree building methods, with sequential jackknifing of outgroup phyla to ensure robustness of phylogenetic affiliations under differing combinations of bacterial genomes. Based on the assessment of nearly 300 phylogenetic tree topologies, we conclude that the continued inclusion of *Epsilonproteobacteria* within the Proteobacteria is not warranted, and that this group should be reassigned to a novel phylum for which we propose the name Epsilonbacteraeota (phyl. nov.). We further recommend the reclassification of the order *Desulfurellales* (*Deltaproteobacteria*) to a novel class within this phylum and a number of subordinate changes to ensure consistency with the genome-based phylogeny. Phylogenomic analysis of 658 genomes belonging to the newly proposed Epsilonbacteraeota suggests that the ancestor of this phylum was an autotrophic, motile, thermophilic chemolithotroph that likely assimilated nitrogen from ammonium taken up from the environment or generated from environmental nitrate and nitrite by employing a variety of functional redox modules. The emergence of chemoorganoheterotrophic lifestyles in several Epsilonbacteraeota families is the result of multiple independent losses of various ancestral chemolithoautotrophic pathways. Our proposed reclassification of this group resolves an important anomaly in bacterial systematics and ensures that the taxonomy of Proteobacteria remains robust, specifically as genome-based taxonomies become more common.

## Introduction

The *Epsilonproteobacteria* were first described in the early 1990s as the fifth subclass of the Proteobacteria (Tenover et al., 1992) and subsequently assigned class status within this phylum (Garrity et al., 2005). The group is widely known for its pathogenic genera *Campylobacter*, *Helicobacter* and, to a lesser extent *Arcobacter*. However, other members of this class are known to play ecologically important roles across a diverse range of environments in which they thrive as mesophiles or moderate thermophiles (Nakagawa and Takaki, 2009). *Epsilonproteobacteria* are important chemolithotrophic primary producers in deep-sea hydrothermal vent systems, where they are often the dominant bacterial lineage in vent plumes and deposits (Huber et al., 2010; Flores et al., 2011), and surrounding microbial mats (Moussard et al., 2006; Opatkiewicz et al., 2009; Rassa et al., 2009). On vent chimneys, *Epsilonproteobacteria* can account for up to 85% of the microbial biomass (Nakagawa et al., 2006). Their metabolic capacity to perform sulfur oxidation coupled to N-oxide reduction while fixing carbon via the reverse TCA cycle (Hügler et al., 2005; Campbell et al., 2009) enables them to be early colonizers of uninhabited vent ecosystems (Alain et al., 2004; Campbell et al., 2006; Gulmann et al., 2015). Non-pathogenic relatives of *Campylobacter* such as *Sulfurospirillum* and *Thiovulum* are often detected in sulfide-rich sediments while others show an affinity for hydrocarbon-rich environments (Hubert et al., 2012). Host-association is also common in this class: *Campylobacter*, *Helicobacter*, and some *Arcobacter* species are known opportunistic pathogens of vertebrates while members of the *Caminibacter*, *Nautilia*, and *Sulfurospirillum* have been reported in association with deep-sea hydrothermal vent fauna. Recent metatranscriptomic data highlighted the role of *Sulfurimonas*-like bacteria in hydrogenase-driven sulfur oxidation and denitrification on the gills of a deep vent sea snail (Sanders et al., 2013).

While the *Epsilonproteobacteria* constitute a stable monophyletic group within the bacterial tree of life, a number of studies suggest that they do not reproducibly affiliate with other Proteobacteria, with the exception of the *Desulfurellales*, which are presently classified as an order of the *Deltaproteobacteria* (see below). Early conserved marker gene-based studies showing *Epsilonproteobacteria* branching immediately basal to other Proteobacteria often had inadequate outgroups and/or bootstrap values supporting this placement (Tenover et al., 1992; Trust et al., 1994; Eisen, 1995; Ludwig et al., 1995; Klenk et al., 1999), while others did not resolve this association at all (Gupta, 2000; Reysenbach et al., 2000; Sheridan et al., 2003; Yarza et al., 2014). More recent phylogenomic evidence based on multiple marker proteins and greater outgroup representation have largely failed to recover the *Epsilonproteobacteria* as reproducibly monophyletic with the rest of the Proteobacteria, further suggesting that taxonomic revision is required at the phylum level (Wu et al., 2009; Di Rienzi et al., 2013; Dodsworth et al., 2013; McLean et al., 2013; Rinke et al., 2013; Zhang and Sievert, 2014; Hug et al., 2016; Yeoh et al., 2016). The class *Epsilonproteobacteria* currently comprises two orders, *Campylobacterales* and *Nautiliales*, encompassing a number of species with ambiguous placement. Particularly problematic are the genera *Nitratifractor* and *Nitratiruptor*. The SILVA and LPSN taxonomies (Quast et al., 2013; Parte, 2014; Yilmaz et al., 2014) currently list these organisms as members of the *Nautiliaceae*, but this classification is not universally accepted (Nakagawa and Takai, 2014) and phylogenetic evidence suggests that they may represent novel families (Nakagawa and Takaki, 2009; Anderson et al., 2011). In a review of the *Epsilonproteobacteria*, Campbell et al. (2006) used the placeholder family *Thiovulgaceae* to group the *Nitratifractor* with *Sulfurovum* and *Sulfurimonas*, and the family *Nitratiruptoraceae* to describe the divergent nature of *Nitratiruptor* from other families. An identical taxonomy was proposed with recent phylogenomic evidence (Zhang and Sievert, 2014), which also revealed a stable monophyletic clade of *Epsilonproteobacteria* with the deltaproteobacterial genus *Hippea*, a member of the order *Desulfurellales*. Both genera belonging to this order, *Desulfurella* and *Hippea*, have low 16S rRNA gene sequence identity to other members of the *Deltaproteobacteria* and frequently form a clade with members of the *Epsilonproteobacteria* (Haddad et al., 1995; Moyer et al., 1996; Miroshnichenko et al., 2002; Kersters et al., 2006; Florentino et al., 2016), suggesting that they should be transferred from the *Deltaproteobacteria* to this group.

The widespread adoption of high-throughput sequencing technologies has resulted in the number of sequenced genomes from bacteria exceeding 70,000 in recent years (Mukherjee et al., 2017)^1^. Additionally, advances in obtaining high-quality draft genomes from metagenomic data (population genomes; Wrighton et al., 2012; Albertsen et al., 2013) and single cells (Marcy et al., 2007; Rinke et al., 2013) greatly augments genomic coverage of microbial diversity and provides the opportunity to supplant the 16S rRNA gene as the basis for microbial classification. Here, we report a phylogenomic characterization of 624 publicly available *Epsilonproteobacteria* and *Desulfurellales* isolate genomes supplemented with 33 *Epsilonproteobacteria* population genomes. As part of this study, we also sequenced a near-complete genome of *Hydrogenimonas thermophila*, and analyzed three partial genomes of single cells belonging to the genus *Thioreductor*. Based on our results, we propose reclassifying the *Epsilonproteobacteria* and *Desulfurellales* as a new phylum, the Epsilonbacteraeota (phyl. nov.), together with a number of subordinate changes and additions at the order and family levels.

## Materials and Methods

### Genome Data

An ingroup comprising 619 *Epsilonproteobacteria*, four *Hippea* species and *Desulfurella acetivorans* were obtained from NCBI RefSeq and GenBank (Supplementary Table S1), and 33 *Epsilonproteobacteria* population genomes (Supplementary Table S2) were recovered from public metagenomic datasets^2^. The genome of *H. thermophila* was sequenced using the Illumina HiSeq 2500 platform (2 × 150 bp chemistry). Raw sequence data (2.4 M reads) were quality filtered using trimmomatic v0.33 (Bolger et al., 2014) in paired end mode, requiring an average quality score of *Q* ≥ 20 over a sliding window of four bases, and a minimum sequence length of 36 nucleotides. A draft genome was assembled using SPAdes v3.8.1 (Bankevich et al., 2012) with a kmer size range of 35–75 (step size = 4) and automatic coverage cutoff. The genome was then scaffolded using FinishM v0.0.9^3^, and scaffolds assessed for assembly errors using RefineM v0.0.13^4^.

Three partial *Thioreductor* genomes were obtained by single cell genome sequencing (Supplementary Table S2). Raw sequence data (41 M reads) were quality filtered as per *H. thermophila*. Quality-filtered sequences were digitally normalized using khmer v2.0 (Crusoe et al., 2015) using the default two-pass approach. Normalized sequences were assembled using SPAdes, and the resulting contigs were scaffolded and refined using RefineM and FinishM as for *H. thermophila*. The taxonomic identity of each *Thioreductor* genome was confirmed by screening high-quality reads for 16S rRNA gene sequence fragments using GraftM^5^. Putative 16S rRNA gene fragments were aligned using the SINA web aligner (Pruesse et al., 2012) and inserted into the SILVA SSU non-redundant database v123.1 using the parsimony insertion tool in ARB.

An outgroup of 4,072 publicly available genomes representing unique species of 24 bacterial phyla were also obtained from NCBI. Completeness and contamination of all genomes was estimated using CheckM v1.0.6 with default settings (Parks et al., 2015).

### Phylogenetic Inference

Ingroups for phylogenetic analyses were selected from the 653 *Epsilonproteobacteria* (including *H. thermophila* and the 33 population genomes) and five *Desulfurellales* genomes. The three partial *Thioreductor* genomes were only included in a reduced concatenated gene analysis due to their low estimated completeness (see below). To resolve the placement of the ingroup in the bacterial domain, 98 ingroup genomes representative at the species-level were selected and combined with the 4,072 outgroup genomes described above. Phylogenetic inference was performed on the 4,170 genomes using a concatenation of 120 conserved protein marker sequences (Ormerod et al., 2016). Protein sequences in each genome were identified and aligned to reference alignments using hmmer v3.1 (Eddy, 1998). Aligned markers were then concatenated and poorly aligned regions removed using Gblocks v0.91b (Castresana, 2000; Talavera and Castresana, 2007).

Maximum likelihood inference of the multiple sequence alignment was performed using the Jones-Taylor-Thornton (JTT), Whelan and Goldman (WAG), and Le and Gascuel (LG) models for amino acid evolution with gamma distributed rate heterogeneity (+Γ) (Jones et al., 1992; Whelan and Goldman, 2001; Le and Gascuel, 2008) implemented in FastTree v2.1.9 (Price et al., 2009). Neighbor joining (NJ) was performed using the Jukes-Cantor and Kimura distance corrections, and with an uncorrected distance matrix implemented in Clearcut v1.0.9 (Sheneman et al., 2006). Under each model/correction, tree building was performed with all sequences included, then once with each phylum or singleton lineage removed, with the exception of Proteobacteria and ingroup genomes (a total of 186 trees). All trees were bootstrap-resampled 100 times to assess the stability of tree topologies. Robustness and reproducibility of the tree topology and association between the *Epsilonproteobacteria*, *Desulfurellales*, and Proteobacteria was assessed by manual examination of all tree topologies in ARB (Ludwig et al., 2004).

To resolve the internal structure of Epsilonbacteraeota relationships, a slightly larger data set of 110 ingroup genomes were selected (106 *Epsilonproteobacteria*, four *Desulfurellales*) and maximum likelihood inference performed using RAxML v8.1.11 (Stamatakis, 2014). An outgroup of 10 genomes representing six bacterial phyla, including Proteobacteria and Aquificae was used to root this tree. Phylogenetic inference was performed with the WAG+Γ model and 100 bootstrap resamples. To further evaluate the robustness of Epsilonbacteraeota relationships, single gene trees were constructed using FastTree with the WAG+Γ model on the individual protein alignments and tree topologies compared to that of the concatenated alignment using the phytools and ape packages in the R software environment (Paradis et al., 2004; Revell, 2012; R Core Team, 2016).

Manual inspection of the three partial *Thioreductor* genomes identified 14 protein families common to all three. Phylogenetic analysis of *Thioreductor* was performed using the above set of 110 ingroup genomes and associated outgroup, using only these 14 protein markers. Phylogenetic inference was performed using RAxML as described above. To assess the placement of species for which genome data is not available, 16S rRNA gene analysis was performed. Epsilonbacteraeota sequences were obtained from the SILVA Living Tree Project v123 (Yilmaz et al., 2014). As this database does not possess a representative for the genus *Thiovulum*, a 16S rRNA sequence for this lineage was obtained from NCBI GenBank. Full length 16S rRNA gene sequences of *Thiofractor thiocaminus*, *Candidatus* Thioturbo danicus, *Cetia pacifica*, and *Thioreductor* species were aligned using the SINA web aligner (Pruesse et al., 2012). An outgroup comprising members of the Proteobacteria, Aquificae, and four other phyla was used to root the tree. The sequence alignment was masked using the LTP 50% SSU conservation filter prior to tree construction. Phylogenetic inference of the masked alignment was performed using RAxML with the general time reversible model with gamma distributed rate heterogeneity and 1,000 bootstrap resamples. Short sequences (<1,000 bp) belonging to *Candidatus* Thioturbo danicus and *Thioreductor* sp. Shim25-G were inserted into the resulting topology using the ARB parsimony insert tool. All tree figures were edited for publication in Inkscape v0.48.

### Sequence Similarity Comparisons

In order to compare our taxonomic proposals to previously proposed sequence similarity-based thresholds for taxonomic ranking, we performed 16S rRNA gene sequence and amino acid identity (AAI) comparisons between members of reclassified or newly proposed families. 16S rRNA gene sequences belonging to *Epsilonproteobacteria* and *Desulfurellales* type strains were extracted from the SILVA Living Tree Project v123 database. Sequences were aligned and hypervariable regions removed using the Lane mask (Lane, 1991). Pairwise sequence distances were calculated for 16S rRNA sequences belonging to the same family but different genera using mothur v1.39.1 (Schloss et al., 2009). AAIs of all best bi-directional diamond (Buchfink et al., 2015) hits between pairwise comparisons of the 110 ingroup genomes were made using CompareM v0.0.21^6^. AAI scores were obtained for genome pairs belonging to the same family, but different genera. Sequence similarity results for each family were visualized using R and compared to previously proposed taxonomic rank boundaries (Konstantinidis and Tiedje, 2005; Yarza et al., 2014).

### Functional Profiling of Epsilonbacteraeota

Functional gene predictions for all Epsilonbacteraeota genomes were performed using Prodigal v2.6.3 (Hyatt et al., 2010). Amino acid translations of predicted genes were annotated using diamond v0.8.10.72 (Buchfink et al., 2015) against the Uniref 100 database (downloaded October 2015) and the accessions of target sequences mapped to their KEGG Orthology (KO) group. Annotations were transformed into an abundance matrix using a custom perl script and principal component analysis was performed using the R package vegan v2.3 (Oksanen et al., 2016). Genomes were partitioned into host-associated or ‘environmental’ and indicator analysis was performed using the package indicspecies (De Cáceres and Legendre, 2009; De Cáceres et al., 2011). KO groups that were significantly associated with either the host-associated or environmental lifestyle were grouped into their functional pathway, and fitted to the PCA ordination using the envfit function in vegan. Additional annotation of hydrogenase enzymes was performed using BLAST (Altschul et al., 1990) against a manually curated database (Greening et al., 2016). Homologous sequences were defined as greater than 30% AAI over at least 70% of the target protein length. Annotation of the reference proteins ACM93230, ACM93747, and ACM93557 of the pathway proposed to facilitate nitrite reduction to ammonium in *Nautilia profundicola* (Campbell et al., 2009; Hanson et al., 2013) was performed with the same BLAST parameters as for hydrogenases.

Phylogenetic analyses of genes involved in carbon fixation, nitrogen and sulfur cycling, and flagella structure and formation were performed using mingle v0.0.18^7^. Protein markers for marker genes (Supplementary Table S3) were downloaded from UniProt and used for initial homolog discovery against the Genome Taxonomy Database (GTDB)^8^. Putative protein homologs were manually inspected for false positive matches and genes below the identity threshold or with inconsistent annotations were removed. Putative citrate lyase alpha/beta subunits sequences were also removed if a homolog of each protein in the pair was not detected in a given genome to ensure paralogs were not being directly compared. A similar strategy was applied to the Sox thiosulfate oxidation proteins (SoxA and SoxB). For each data set, protein sequences were aligned using MAFFT v7.221 using the L-INS-i algorithm (Katoh et al., 2002; Katoh and Standley, 2013). The alignment was then masked using Gblocks and phylogenetic inference performed with RAxML as described above.

## Results and Discussion

### Genome Data

A total of 619 *Epsilonproteobacteria* and five *Desulfurellales* genomes were obtained from RefSeq version 76 and GenBank version 213 (Supplementary Table S1). Genomes were assessed for completeness and contamination by scoring the presence of conserved single-copy marker genes within each genome using CheckM (Parks et al., 2015). The median estimated genome completeness for this dataset is 99.4% and the minimum is 81.9%. Genomes were estimated to be less than 10% contaminated, with all but eight under 5% (Supplementary Table S1). The taxonomic annotation of the type strain *Campylobacter geochelonis* (GCA_900063025.1) was manually modified as the NCBI record for this genome incorrectly labels it as *C. fetus* (Piccirillo et al., 2016). Thirty-three draft population genomes (median completeness 93.8%, contamination 1.1%) belonging to the *Epsilonproteobacteria* were recovered from publicly available metagenomic data sets as part of a larger study (Parks et al., submitted) and included in our analysis. In addition to the public genomes, we sequenced the type strain of *H. thermophila*, sole representative of the genus *Hydrogenimonas* (Takai et al., 2004) and three single cells belonging to the genus *Thioreductor* (Supplementary Table S2). For *H. thermophila*, an Illumina-based assembly produced a draft genome of 96 contigs with a predicted completeness of 99.6 and 1.8% contamination. *Thioreductor* single cells amplifications were assembled into partial genomes with completeness estimates between 27.7 and 36.5%, and with low contamination estimates (0.3–1.2%) (Supplementary Table S2). Owing to their low completeness *Thioreductor* genomes were excluded from the majority of analyses, resulting in an ingroup comprising 658 quality-filtered genomes (119 complete and 539 draft) for comparative analysis. Outgroup genomes broadly representative of the bacterial domain were selected from a total of 60,258 quality controlled reference genomes available from the Genome Taxonomy Database.

### Proposed Genome-Based Taxonomy

**FIGURE 1:**
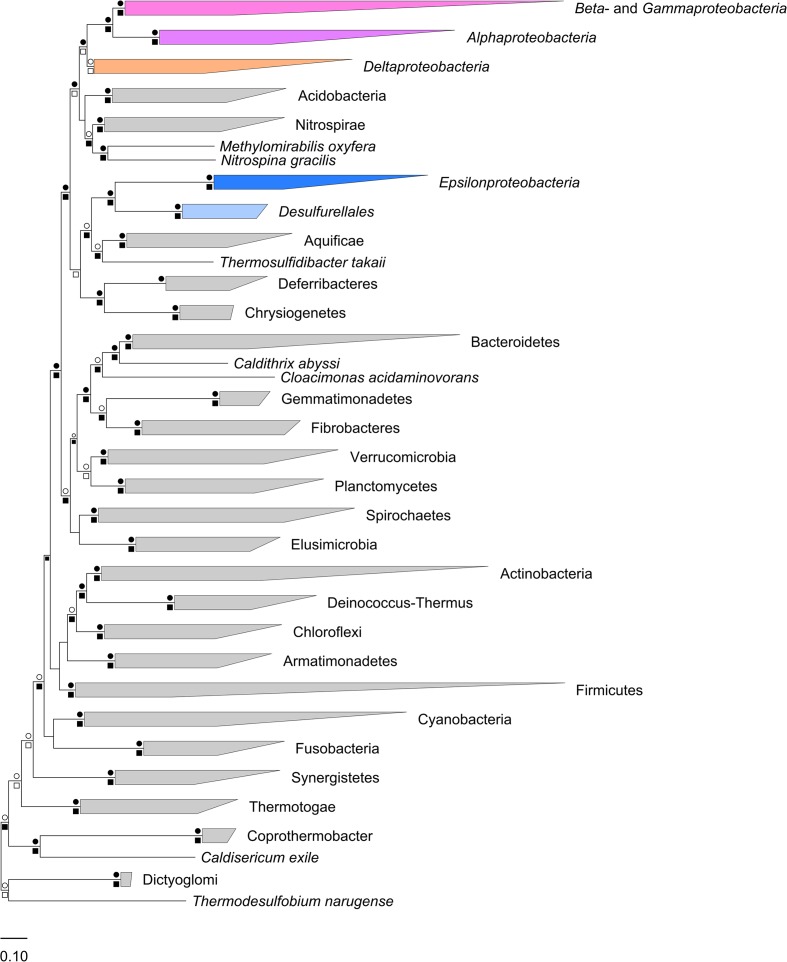
**Maximum likelihood phylogenetic inference (WAG + Γ model) of *Epsilonproteobacteria* and *Desulfurellales* in the context of 4,170 bacterial genomes and based on 120 concatenated protein sequences**. Robustness of the placement of the Epsilonbacteraeota was assessed using bootstrap support and per-phylum jackknifing. Symbols above each branch root (circles) represent bootstrap support of >90% (black) or ≥75% (hollow). Symbols below each root reflect jackknife reproducibility of a node, with black square representing nodes recovered with ≥90% support in ≥90% of tree topologies. Hollow squares indicate ≥75% support in ≥90% of topologies. Under every tree topology explored *Epsilonproteobacteria* and *Desulfurellales* were recovered as a strongly supported monophyletic group with no association to the remaining Proteobacteria. Key findings of tree topologies under alternate tree building models are summarized in **Table 1**.

Phylogenetic affiliation(s) of the ingroup (*Epsilonproteobacteria* and *Desulfurellales*, 98 genomes) to species-level representatives of the outgroup (4,072 genomes) were assessed using two different datasets. The first dataset was a concatenation of 120 single-copy marker proteins (Parks et al., submitted) and the second was a concatenation of the 16S and 23S rRNA gene sequences (Williams et al., 2010; Abby et al., 2012; Kozubal et al., 2013; Guy et al., 2014; Ochoa de Alda et al., 2014; Sen et al., 2014). Note that the 3,144 genomes contributing to the second dataset are a subset of the first as most genome sequences derived from metagenomic data lack complete rRNA gene sequences (Hugenholtz et al., 2016), and is used here primarily to validate the concatenated protein tree. Based on these datasets, phylogenetic trees were inferred using Maximum Likelihood (ML) with the JTT, WAG, and LG models of amino acid substitution (Jones et al., 1992; Whelan and Goldman, 2001; Le and Gascuel, 2008) as well as NJ with Jukes-Cantor and Kimura distance corrections (Jukes and Cantor, 1969; Kimura, 1980). Robustness of tree topologies was analyzed with a combination of bootstrapping and taxon resampling, implemented by removal of one phylum at a time from the outgroup dataset. The consensus of these analyses indicate that the *Epsilonproteobacteria* and *Desulfurellales* are robustly monophyletic and not reproducibly affiliated with any other phyla (**Figure 1** and **Table 1**), which is consistent with recent reports also using concatenated protein markers (Zhang and Sievert, 2014; Hug et al., 2016). The phylum-level jackknife analysis suggests a specific association of the ingroup with the Aquificae, which is also supported by bootstrap resampling of this dataset (**Figure 1**). Tree topologies which suggest a common ancestry between Aquificae and *Epsilonproteobacteria* have been reported for several marker genes (Gruber and Bryant, 1998; Klenk et al., 1999; Iyer et al., 2004); however, this association is often not statistically robust. Phylogenomic evidence suggests that Aquificae genomes have been shaped by extensive lateral gene transfer from lineages including the *Epsilonproteobacteria* (Eveleigh et al., 2013), a phenomenon that might have contributed to the observed association. Importantly, removal of the Aquificae in the jackknife analysis did not affect the apparent separation of the *Epsilonproteobacteria* from the other proteobacterial classes.

**Table 1:** Support of lineage associations under different tree building methods.

Tree method	Model	C-N	C-N-D	C-N-D-A	C-N-P	D-P
ML (FastTree)	WAG + Gamma	^∗^	^∗^	^∗^		
	JTT + Gamma	^∗^	^∗^	^∗^		
	LG + Gamma	^∗^	^∗^	^∗^		
NJ (Clearcut)	No correction^a^	^∗^				
	Jukes-Cantor correction	^∗^	^∗^			
	Kimura correction	^∗^	^∗^			

*Columns refer to collections of orders or phyla which were robustly monophyletic, but not intermingled. Asterisk (^∗^) represents affiliations supported by both bootstrap resampling of the full tree (>90% support), and per-phylum jackknife analysis (>90% bootstrap support in >90% of jackknife trees). No instances were observed where an association was resolved under one criteria but not the other. Letters represent a robustly monophyletic group of genomes, taxonomically classified as C: Campylobacterales, N: *Nautiliales*, D: *Desulfurellales*, A: Aquificae, P: Proteobacteria. Between-group polyphyly was not observed for any permutation of groups. ^a^ The C-N-D affiliation is not supported, so C-N-D-A could not be tested. However, neither C-N-A or D-A affiliations were supported under this tree topology*.

### Proposal for a New Phylum

**FIGURE 2:**
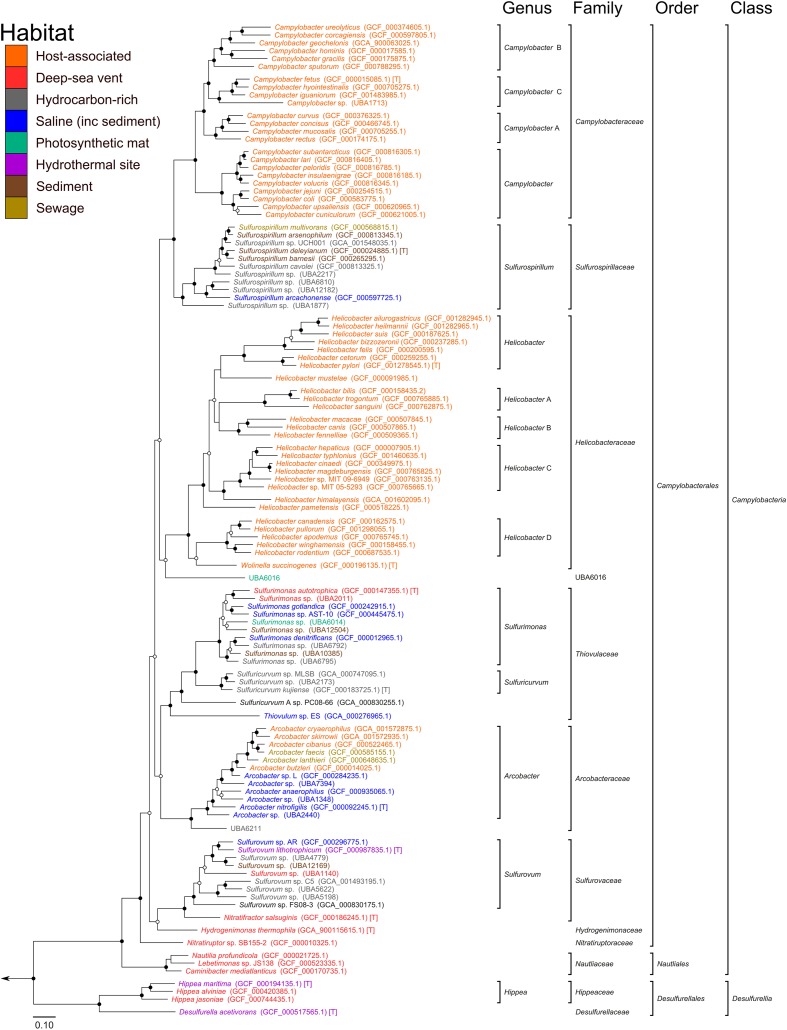
**Phylogenetic analysis of the Epsilonbacteraeota**. Maximum likelihood tree of the Epsilonbacteraeota based on 120 concatenated protein marker sequences using RAxML. Support of internal nodes was calculated using 100 bootstrap iterations, and junctions represent nodes with 100% bootstrap support (solid), and >75% support (hollow). All taxonomic groupings are identical to those in **Figure 1** (calculated with FastTree), although the branching positions of *Helicobacteraceae* and *Sulfurovaceae* differs between the two topologies. [T]: denotes type species.

Based on our phylogenetic analyses, we propose to reclassify the *Epsilonproteobacteria* and *Desulfurellales* as a single phylum called the Epsilonbacteraeota (phyl. nov.), consistent with a recently proposed naming convention for the phylum rank (Oren et al., 2015). The *Epsilonproteobacteria* constitute a class-level lineage within this phylum (**Figure 2** and **Table 2**), but we propose to rename it as *Campylobacteria* (class. nov.) to remove any remaining association with the phylum Proteobacteria. The existing *Epsilonproteobacteria* orders *Campylobacterales* and *Nautiliales* are maintained within this proposed taxonomy as they represent robustly monophyletic groupings and have no direct link to the Proteobacteria in their names (**Figure 2** and Supplementary Figure S1 and Table S4). Similarly, the taxon *Desulfurellales* is retained as the sole order within a new class, the *Desulfurellia* (class. nov.) (**Figure 2** and Supplementary Figure S1). Family and genus level groupings are mostly consistent with existing taxonomic authorities (**Table 2**), and reclassified or newly proposed families fall within proposed guidelines for 16S rRNA gene similarity (Yarza et al., 2014) and AAI (Konstantinidis and Tiedje, 2005) thresholds (Supplementary Figure S2). Proposed changes to family membership are detailed below.

**Table 2:** Taxonomy of the Epsilonbacteraeota compared to existing *Epsilonproteobacteria*.

Class/Order	Family	Genus	Bergey’s	NCBI	SILVA LTP v123^a^	Greengenes 13_5	LPSN


*Campylobacteria*			*Epsilonproteobacteria*	*–*	*–*	*–*	*–*


*Campylobacterales*							


	***Arcobacteraceae***		*Campylobacteraceae*	*–*	*–*	*–*	*–*


		*Arcobacter*					


	*Campylobacteraceae*						


		*Campylobacter*					


	*Helicobacteraceae*						


		*Helicobacter*					


		*Wolinella*					


	*Hydrogenimonaceae*						


		*Hydrogenimonas*					


	***Nitratiruptoraceae***			*Epsilonproteobacteria*	*Nautiliaceae*		*Nautiliaceae*


		*Nitratiruptor*					


	***Sulfurospirillaceae***		*Campylobacteraceae*	*–*	*–*	*–*	*–*


		*Sulfurospirillum*					


	***Sulfurovaceae***			Unclassified	*Helicobacteraceae*/*Nautiliaceae*^b^		*Helicobacteraceae*/*Nautiliaceae*^b^


		*Sulfurovum*					


		*Nitratifractor*					


	***Thiovulaceae***		*Helicobacteraceae*	*–*	*–*	*–*	*–*


		*Thiovulum*					


		*Sulfuricurvum*					


		*Sulfurimonas*					


	Family incertae sedis						


		*Thiofractor*^c^		Unclassified	*Campylobacteraceae*		


		*Candidatus* Thioturbo^c^					


*Nautiliales*			*Campylobacterales*				


	*Nautiliaceae*						


		*Nautilia*					


		*Caminibacter*					


		*Lebetimonas*					


		*Cetia*^c^					


	***Thioreductoraceae***			*Epsilonproteobacteria*	*Nautiliaceae*		*Nautiliales*


		*Thioreductor*^d^					


*Desulfurellia*			*Deltaproteobacteria*	*–*	*–*	*–*	*–*


*Desulfurellales*							


	*Desulfurellaceae*						


		*Desulfurella*					


	***Hippeaceae***			*Desulfurellaceae*	*Desulfurellaceae*		*Desulfurellaceae*


		*Hippea*					

*Current classification for each ranking is provided for commonly used taxonomic schemes comparing the proposed Epsilonbacteraeota (left) to currently accepted taxonomic schemes (right). Empty cells reflects an unchanged classification for Epsilonbacteraeota. Dashed (–) cells represent a classification consistent to Bergey’s in SILVA, Greengenes, or LPSN. Shaded cells represent an entry not recorded under the taxonomic scheme. Genera listed as ‘Unclassified’ in NCBI are recognized as a part of the *Epsilonproteobacteria*, but have no assignment below the class level. ^a^The genera *Thiofractor* and *Sulfuricurvum* are annotated in SILVA SSU taxonomy, but not recorded in LTP. ^b^Genera of this family do not belong to a single family under this taxonomic scheme. ^c^Only 16S rRNA gene sequence data is available for this genus. ^d^*Thioreductor* placement is based on concatenated alignment of 14 protein markers. Novel families proposed in this study are bolded*.

#### Changes to the *Campylobacteraceae*

The currently defined family *Campylobacteraceae*, comprised of the genera *Arcobacter*, *Campylobacter*, and *Sulfurospirillum*, is polyphyletic in the concatenated protein tree (**Figure 2**), although it is monophyletic in the concatenated rRNA gene tree (Supplementary Figure S1). Inspection of individual protein tree topologies revealed that only two of the 120 markers resolve the *Campylobacteraceae* as monophyletic (Supplementary Figure S3). For this reason, we propose to transfer the *Arcobacter* and *Sulfurospirillum* into their own families, the *Arcobacteraceae* (fam. nov.) and *Sulfurospirillaceae* (fam. nov.), respectively. *Campylobacter* is a deeply divergent genus which likely requires reclassification into a number of distinct genera (indicated in **Figure 2** by alphabetical suffixing). Note also that *Dehalospirillum multivorans* and *Geospirillum barnesii* have been formally transferred to the genus *Sulfurospirillum* (Finster et al., 1997; Luijten et al., 2003), and *Thiomicrospira denitrificans* has been reclassified to *Sulfurimonas* (Takai et al., 2006), although these names persist in some online archives.

#### Changes to the *Helicobacteraceae*

The currently defined *Helicobacteraceae* do not form a monophyletic group to the exclusion of the *Campylobacteraceae* and *Arcobacter*; therefore, the genera *Thiovulum*, *Sulfuricurvum*, and *Sulfurimonas* have been removed from the *Helicobacteraceae* into their own family, the *Thiovulaceae* (fam. nov.). Monophyly of the *Helicobacteraceae* is also not robustly supported in the concatenated rRNA gene tree (Supplementary Figure S1). Species in the *Sulfurovum* and *Nitratifractor* genera are either currently unclassified at the family level, or classified into the families *Helicobacteraceae* and *Nautiliaceae*, respectively (**Table 2**). Like the *Campylobacter*, *Helicobacter* is a deeply divergent genus which likely requires reclassification into a number of distinct genera (indicated in **Figure 2** by alphabetical suffixing). *Sulfurovum* and *Nitratifractor* are resolved as a robustly monophyletic group, independent of both the *Helicobacteraceae* and *Nautiliaceae*, for which we propose the name *Sulfurovaceae* (fam. nov., **Figure 2**). However, the concatenated rRNA gene tree does not support the monophyly of the *Sulfurovaceae* (Supplementary Figure S1). Inspection of individual protein trees reveals that the majority of markers (105/120) supports the association (Supplementary Figure S3), as well as the 16S rRNA gene by itself (Supplementary Figure S4). The revised *Helicobacteraceae* family comprises only species in the genera *Helicobacter* and *Wolinella* (**Figure 2**). Note that the genus *Flexispira* (Bryner et al., 1986) is not included in the *Helicobacteraceae* as it is a defunct basonym of *Helicobacter* (Dewhirst et al., 2000; Vandamme and On, 2001).

#### Proposal for New Families

We further propose that the genus *Nitratiruptor* be placed into its own family *Nitratiruptoraceae* (fam. nov.) within the order *Campylobacterales*, a move that, while inconsistent with taxonomic authorities placing this genus in the *Nautiliaceae* (**Table 2**), is supported by 16S rRNA gene-based phylogenies (Nakagawa and Takaki, 2009; Anderson et al., 2011; Nakagawa and Takai, 2014). Analysis of the three partial single cell *Thioreductor* genomes confirms the 16S rRNA-based analysis that this genus is a member of the family *Nautiliaceae* (Supplementary Figures S4, S5). In addition to the proposed revisions in the class *Campylobacteria*, we propose that *Hippea* be transferred to its own family *Hippaceae* (fam. nov.) within the class *Desulfurellia* to reflect the depth of its relationship with the genus *Desulfurella* (**Figure 2**). A complete summary of proposed changes within the Epsilonbacteraeota are provided in Supplementary Table S3.

#### Genera Not Yet Represented by Complete Genome Sequences

There are three published genera presently classified as *Epsilonproteobacteria* for which genomic data are currently unavailable: *Thiofractor*, *Candidatus* Thioturbo, and *Cetia*. These genera are therefore provisionally placed within the Epsilonbacteraeota based on comparative analysis of 16S rRNA gene sequences alone (Supplementary Figure S4); however, their classification may require future revision as genomic information becomes available. *Thiofractor* and *Candidatus* Thioturbo appear to be members of the order *Campylobacterales* consistent with previous findings (Muyzer et al., 2005; Makita et al., 2012), however, neither are clearly resolved into the family level groupings proposed for the Epsilonbacteraeota. *Thiofractor* may constitute its own family and *Candidatus* Thioturbo may be incorporated into the *Arcobacteraceae*, although this latter affiliation is only based on a partial 16S rRNA sequence (Supplementary Figure S4). *Cetia pacifica* clusters robustly within the family *Nautiliaceae*, which may or may not require reclassification of the genus *Caminibacter* (Supplementary Figure S4; Grosche et al., 2015).

### Functional Profiling of Epsilonbacteraeota

**FIGURE 3:**
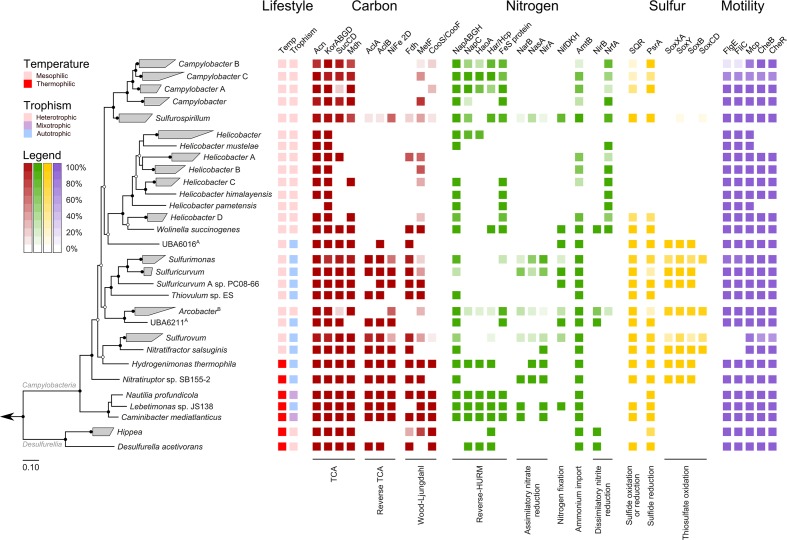
**Habitat and functional annotation of selected Epsilonbacteraeota pathways**. Tree topology is that of **Figure 2**, collapsed at the genus level. Cell intensity reflects the proportion of genomes within a clade that possessed the column function. A: Trophism is inferred from genomic data. B: Although some members of *Arcobacter* are capable of autotrophic growth (discussed in text) all genomes in this study were obtained from heterotrophic species. Column descriptions are as follows: Carbon pathways: aconite hydratase (Acn), 2-oxoglutarate oxidoreductase alpha-, beta-, gamma-, and delta- subunits (KorABGD), succinyl-CoA synthetase alpha and beta subunits (SucCD), malate dehydrogenase (Mdh), ATP citrate lyase alpha subunit (AclA) and beta subunit (AclB), [NiFe] hydrogenase family 2D (NiFe 2D), formate dehydrogenase (Fdh), methylenetetrahydrofolate reductase (MetF) and carbon-monoxide dehydrogenase catalytic and iron sulfur subunits (CooS/CooF). Nitrogen pathways: periplasmic nitrate reductase components NapA, NapB, NapG, and NapH (NapABGH), periplasmic nitrate reductase c-type cytochrome (NapC), hydroxylamine oxidoreductase (HaoA), hydroxylamine reductase/hybrid cluster protein (Har/Hcp), predicted reductive Fe-S protein (FeS protein), ferredoxin-nitrate reductase (NarB), assimilatory nitrate reductase (catalytic subunit, NasA), ferredoxin-nitrite reductase (NirA), nitrogenase molybdenum-iron (alpha- and beta-chains) and iron protein (NifDKH), ammonium transporter (AmtB), nitrite reductase (NADH) large subunit (NirB), cytochrome c nitrite reductase (NrfA). Sulfur pathways: sulfide:quinone oxidoreductase (SQR), polysulfide reductase chain A (PsrA), thiosulfate oxidating Sox proteins (SoxXA, SoxY, SoxB, SoxCD). Motility: flagellar hook protein (FlgE), flagellin (FliC), methyl-accepting chemotaxis protein (Mcp), chemotaxis response regulator (CheB), chemotaxis methyltransferase (CheR).

**FIGURE 4:**
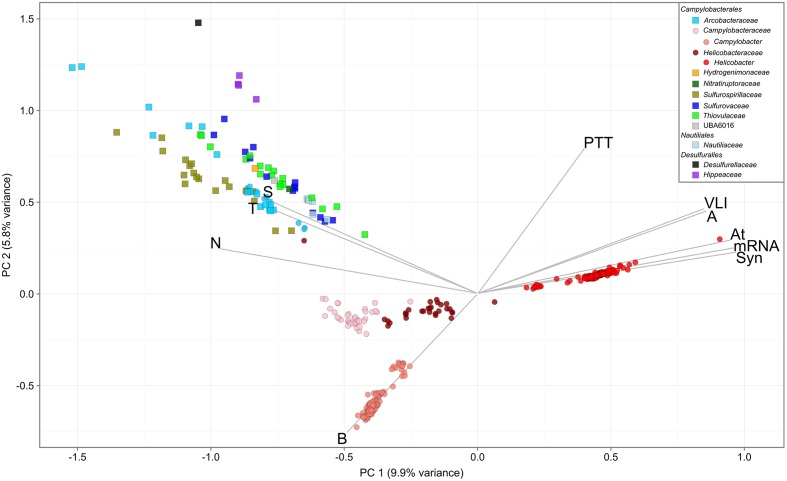
**Principal component analysis of Epsilonbacteraeota gene annotations**. Analysis was performed using the KEGG Orthology (KO) annotation table. Circles denote host-associated individuals, and squares those from other environments. Genomes are colored by family, with exceptions of the *Campylobacteraceae* and *Helicobacteraceae*, which are separated based on suffixing described in text. Vectors represent pathway level aggregations of indicator KOs fitted to the ordination using the enfit function in vegan. Only vectors with *R* > 0.75 are displayed for clarity. Pathways are as follows: A, arginine biosynthesis; At, atrazine degradation; B, biotin metabolism; N, nitrogen metabolism; PTT, phenylalanine, tyrosine, and tryptophan biosynthesis; S, sulfur metabolism; Syn, synthesis and degradation of ketone bodies; T, two-component system; VLI, valine, leucine, and isoleucine degradation.

Overlaying published phenotypic information of cultured Epsilonbacteraeota on the genome-based phylogeny suggests that the ancestor of this phylum was autotrophic and thermophilic (**Figure 3**). Mesophily arose later in the *Campylobacterales*, and heterotrophic growth appears to have arisen independently in the *Campylobacterales* and *Desulfurellia* (**Figure 3**). To quantify the extent to which taxonomy reflects functional variation amongst Epsilonbacteraeota, we performed PERMANOVA to quantify the contribution of predicted functional profiles using KO. The largest source of variation was taxonomy (family; *R* = 0.68, genus; *R* = 0.70, *p* = 0.001), followed by habitat (*R* = 0.28, *p* = 0.001), indicating that while vertical inheritance is a powerful predictor of functional capacity, a large portion of variation is not captured by this process and likely reflects habitat-specific adaptation. This variation was also reflected in PCA analysis of the functional profiles, where heterotrophic *Campylobacter* and *Helicobacter* genomes were clearly separate from other Epsilonbacteraeota (**Figure 4**). Furthermore, there was pronounced separation within these genera by functional PCA consistent with the suggestion to classify both *Campylobacter* and *Helicobacter* into multiple genera (**Figure 2**). To elucidate the metabolic components driving this separation, indicator analysis (De Cáceres and Legendre, 2009; De Cáceres et al., 2011) was applied to the gene annotation table to highlight key features responsible for functional divergence.

Genes involved in carbon and nitrogen fixation, assimilatory nitrate and nitrite reduction, thiosulfate oxidation and polysulfide reduction were significantly associated with the environmental ecotype indicating that carbon, nitrogen, and sulfur cycling are primary drivers of functional divergence in environmental Epsilonbacteraeota. These results were visually apparent when aligning functional genes against the concatenated protein phylogeny (**Figure 3**). Host-associated Epsilonbacteraeota were defined primarily by the absence of these functions, although consistent with their lifestyle the antimicrobial peptide resistance mechanism YejABEF (Wang et al., 2016) was a key signature of host-association. The separation of *Helicobacter* (containing the type species, *H. pylori*) from *Helicobacter* A to D was driven by the presence of genes involved in osmoprotectant and heme transport (Kappes et al., 1999; Létoffé et al., 2006), as well as the CAG pathogenicity island in *H. pylori*. Similarly, *Campylobacter* differentiation was in part due to the presence of additional transporters involved in lipopolysaccharide and capsular polysaccharide transport. These findings suggest that the transition toward a host-associated lifestyle in these lineages has occurred in a two-step manner. First, the loss of ‘environmental’ functions associated with the initial transition to host-association in *Campylobacter* and *Helicobacter*, followed by gene acquisition to enhance host adaptation. The distinct phylogenetic and functional differences within these genera suggest that future revision of their taxonomy may be warranted.

#### Carbon Metabolism

Analyzing the carbon metabolism of Epsilonbacteraeota revealed a complete tricarboxylic acid (TCA) cycle in all genomes with the exception of *Helicobacter* (**Figure 3**), which functions as a directed pathway producing succinate and α-ketoglutarate in this genus (Pitson et al., 1999). Phylogenetic analysis of the marker protein, 2-oxoglutarate oxidoreductase (KorA), support the TCA cycle being vertically inherited throughout the Epsilonbacteraeota (Supplementary Figure S6). Most genera also encode ATP citrate lyase (AclA and B, **Figure 3**) indicating the ability to perform autotrophic carbon fixation via reductive TCA (rTCA; Buchanan and Arnon, 1990). The presence of this enzyme coincides with phenotypically established autotrophy (Hügler et al., 2005; Campbell et al., 2006), and genera which have lost the ability to fix carbon have adopted an exclusively heterotrophic lifestyle in carbon rich habitats (**Figure 4**) with the exception of a number of autotrophic *Arcobacter* strains (Gevertz et al., 2000; Wirsen et al., 2002) for which genomes are not currently available. Phylogenetic inference of both citrate lyase subunits indicates a monophyletic history of carbon fixation in the Epsilonbacteraeota with independent losses of this capability inferred in the *Campylobacter*, *Helicobacteraceae*, and *Arcobacter* (**Figure 3**). Citrate lyase was detected in a population genome basal to the *Arcobacter* genus (UBA6211, **Figure 3**) which, combined with previous observation of citrate lyase activity and the operation of the rTCA in *Arcobacter* (Hügler et al., 2005), suggests that carbon fixation was present in the common ancestor of this genus. Vertical inheritance was also observed in Epsilonbacteraeota homologs of the cytosolic [NiFe] hydrogenase group 2D, which is hypothesized to provide reducing power for the rTCA (Brugna-Guiral et al., 2003; Greening et al., 2016).

No other complete carbon fixation pathways were identified in the genomes. Formate dehydrogenase is widely distributed in many autotrophs (Schuchmann and Müller, 2014) and was common in hydrothermal vent-associated lineages (**Figures 2**, **3**), many of which are known to oxidize formate (Macy et al., 1996; Campbell et al., 2006; Grote et al., 2012; Grosche et al., 2015; Florentino et al., 2016). A recent biochemical analysis of hydrothermal vents demonstrated that abiotic processes sequester available carbon dioxide as formate (McDermott et al., 2015), and the presence of formate dehydrogenase may provide the ability to couple respiration with the creation of carbon dioxide to fuel rTCA. The Wood-Ljüngdahl (WL) pathway is more energetically favorable than the rTCA but requires strict anoxic conditions to operate (Berg, 2011), which may be incompatible with the microaerobic growth of many vent-associated Epsilonbacteraeota (Campbell et al., 2006). Monophyletic distribution of the WL-specific marker methylene-tetrahydrofolate reductase (MetF, **Figure 3** and Supplementary Figure S7) amongst the *Campylobacteria* suggests that this pathway was an ancestral trait which has been supplanted by the rTCA, possibly in response to the adoption of (micro)aerobic habitats. Consistent with previous analyses (Hügler et al., 2005, 2011; Takai et al., 2005; Wright et al., 2013), RuBisCO (ribulose-1,5-bisphosphate carboxylase/oxygenase) was not detected in any genome, and the rTCA remains the only known means for carbon fixation amongst Epsilonbacteraeota. The pattern of auto- and heterotrophic lifestyles within the Epsilonbacteraeota suggests that autotrophy is an ancestral trait, punctuated by recent adaptations to heterotrophy mostly in host-associated habitats. Additionally, the Epsilonbacteraeota and Aquificae citrate lyase proteins are robustly monophyletic (Supplementary Figure S8), consistent with the inference that carbon fixation in the Epsilonbacteraeota is descended from an ancestral Aquificae phenotype (Braakman and Smith, 2012).

#### Nitrogen Metabolism

Anaerobic respiration using nitrate as an electron acceptor is widespread throughout the Epsilonbacteraeota, as evidenced by the presence of soluble periplasmic nitrate reductase (NapA) in most genera (**Figure 3**), and membrane-bound nitrate reductase (NarG) in *Desulfurella* (Vetriani et al., 2014). The NAP system of many *Campylobacteria* lack the NapC protein (Simon et al., 2003; Sievert et al., 2008; Kern and Simon, 2009) and where a homolog is present, it is proposed to act as the quinol-oxidizing protein component of a reversely operating hydroxylamine:quinone redox module. This pathway operates as a quinone-reducing module in ammonia-oxidizing bacteria and Thaumarchaeota, and has recently been proposed in *N. profundicola* (Klotz and Stein, 2008; Campbell et al., 2009; Hanson et al., 2013; Simon and Klotz, 2013; Kozlowski et al., 2016). As a component of an ammonification pathway, this module produces ammonium by reduction of nitrite either directly (analogous to the NrfHA ammonification module) or via a hydroxylamine intermediate, which is thought to be transported to the cytoplasm and further reduced to ammonium by a putative hydroxylamine reductase (Har/Hcp), powered by an Fe-S ferredoxin (Hanson et al., 2013). While hydroxylamine reduction to ammonium and nitrate-dependent expression of genes encoding the entire pathway has, so far, only been demonstrated in *N. profundicola*, genes for this pathway are present in *Campylobacter concisus*, *C. curvus*, and *C. fetus* (Hanson et al., 2013). We further identified the complete inventory for this proposed pathway in the remaining *Nautiliales* genomes, as well as *Campylobacter mucosalis*, *C. geochelonis*, *C. gracilis*, *C. hyointestinalis*, and *C. iguaniorum* (**Figure 3**). Furthermore, *H. thermophila* and *D. acetivorans* lack only the predicted Fe-S ferredoxin of this pathway, but as hydroxylamine reductase is known to obtain reductant from other oxidoreductases (Pino et al., 2006), it is possible that these organisms are also capable of ammonification using a hydroxylamine intermediate. The NapC homolog and HaoA proteins are homologous to NrfH and NrfA, respectively (Bergmann et al., 2005; Kern et al., 2011a; Simon and Klotz, 2013) and differ mainly by the infidelity of the HaoA oligomer as an ammonium-producing nitrite reductase. These homologs facilitate incomplete electron transfer and leak hydroxylamine due to looser redox coupling with the NapC homolog compared to that of the NrfA-NrfH module (Simon and Klotz, 2013).

Phylogenetic inference of the HaoA and Har/Hcp proteins suggest that this more complex nitrite ammonification pathway is monophyletic and ancestral to the Epsilonbacteraeota, despite widespread partial or complete loss of the pathway in the group, and putative lateral transfers of component genes from Epsilonbacteraeota donors to other bacteria (Supplementary Figure S9). Most Epsilonbacteraeota have multiple potential mechanisms for obtaining fixed nitrogen and the acquisition of chemoorganotrophic catabolic machinery, which provide cells with higher and more reliable reducing power, likely facilitated the functional replacement of the HaoA-type with the NrfA-type nitrite reductase. The majority of extant autotrophic Epsilonbacteraeota possess one or more ammonification pathways including dinitrogen reduction based on nitrogenase and/or a variety of pathways based on the reduction of nitrate (**Figure 3**). Annotation of the *Thiovulum* sp. ES genome did not reveal any ammonification inventory; nevertheless and consistent with its original annotation (Marshall et al., 2012), we detected the gene encoding an ammonium transporter (AmtB) in this lineage, shared with most other Epsilonbacteraeota (**Figure 3**).

Pentaheme cytochrome *c* nitrite reductase (NrfA) is found almost exclusively in heterotrophic *Campylobacteria* (**Figure 3**) and is known to provide protection against nitrosative stress to *Campylobacter* and *Wolinella* in their natural environments (Pittman et al., 2007; Kern et al., 2011b). This protein may also serve the same function in the majority of *Helicobacter* species, although this has not been well studied since the model species, *H. pylori*, lacks NrfA and has alternative mechanisms for coping with nitrosative stress (Flint et al., 2016). In contrast to the concatenated protein-based monophyly of the heterotrophs (**Figure 3**), phylogenetic inference of NrfA reveals at least two independent clades, the first comprising *Sulfurospirillum*, *Wolinella* and some *Arcobacter*, and the second *Campylobacter* and *Helicobacter* (Supplementary Figure S10). It is likely at least one of these clusters is the result of lateral gene transfer, which we predict to be the *Campylobacter* and *Helicobacter* clade as members of these two genera are intermingled indicating multiple lateral transfer events (Supplementary Figure S10), possibly the result of selective pressure exerted by the host immune system (Kern et al., 2011a; Kassem et al., 2012).

#### Sulfur Metabolism

Anaerobic respiration of Epsilonbacteraeota frequently occurs through the reduction of sulfur compounds, and is a major source of primary production in some environments (Wright et al., 2013). Sulfide-oxidizing sulfide:quinone oxidoreductase (SQR) catalyzes the bidirectional oxidation of hydrogen sulfide to polysulfide, and this gene is inferred to be abundant in environments where Epsilonbacteraeota (*Epsilonproteobacteria*) are dominant members (Wright et al., 2013; Rossmassler et al., 2016). Consistent with this inference, we observed SQR genes in most Epsilonbacteraeota lineages (**Figure 3**). In contrast to the monophyletic origins of the carbon and most nitrogen cycling proteins described above, phylogenetic analysis of SQR reveals extensive polyphyly of Epsilonbacteraeota homologs, including a putative lateral transfer from a Bacteroidetes donor to the *Nautilaceae* ancestor (Supplementary Figure S11). An additional enzyme involved in polysulfide reduction, polysulfide reductase (PsrA) was also common in the Epsilonbacteraeota (**Figure 3**) and possessed polyphyletic origins (Supplementary Figure S12). These findings suggest that unlike nitrate reduction, sulfur reduction has been acquired in the Epsilonbacteraeota in response to immediate environmental needs. Polysulfide can be formed abiotically under hydrothermal vent conditions (Rushdi and Simoneit, 2005; Boyd and Druschel, 2013) and, similar to formate, presents a resource that early colonizing Epsilonbacteraeota may exploit. It is possible that the acquisition of SQR represents a means of simultaneously detoxifying hydrogen sulfide, and utilizing it as an electron donor, as *Epsilonproteobacteria* have been seen to bloom in response to hydrogen sulfide releases on the African shelf (Lavik et al., 2009).

Oxidation of sulfur is also a well known trait of *Epsilonproteobacteria*, although it is only reported in a handful of genera (Campbell et al., 2006). *Sulfurimonas*-like organisms have been suggested as the primary sulfur-oxidisers in deep-sea vent ecosystems (Akerman et al., 2013) and consistent with this observation, *Sulfurimonas* was one of the few Epsilonbacteraeota genera to possess the complete sulfur oxidation (*sox*) pathway (**Figure 3**). The absence of SoxC, and drastic reduction in electron liberation (Friedrich et al., 2005), in most genera suggests that sulfur oxidation is not the primary means of electron generation amongst Epsilonbacteraeota, with hydrogenases likely a more productive source of reducing power. The evolutionary history of sox in the Epsilonbacteraeota is convoluted. Consistent with previous analysis of *sox* genes (Meyer et al., 2007; Ghosh et al., 2009), SoxA and SoxB homologs in non-*Arcobacter* species shared a common ancestor with Aquificae, while *Arcobacter* acquired a complete *sox* cassette through lateral gene transfer with *Gammaproteobacteria* (Supplementary Figure S13). However, homologs of SoxC are monophyletic in the Epsilonbacteraeota, a finding also supported by previously literature (Ghosh et al., 2009). These data suggest that after acquiring the ability to oxidize thiosulfate independently of other Epsilonbacteraeota, an ancestor of *Arcobacter* introduced soxCD to *Sulfurimonas* and the *Sulfurovaceae*.

#### Motility

The majority of Epsilonbacteraeota possess uni- or bipolar flagella and are highly motile, with *Thiovulum majus* capable of achieving a speed of over 600 microns per second, or 2 m per hour (Garcia-Pichel, 1989). The flagella of Epsilonbacteraeota differ from that of other bacteria in several respects. For example, the C-ring of model *Campylobacter jejuni* and *Helicobacter hepaticus* differs structurally from that of other bacteria (Chen et al., 2011), and recent knock-out experiments identified six novel genes exclusive to flagella activity in Epsilonbacteraeota (Gao et al., 2014). Given the novelty of described Epsilonbacteraeota flagella, we predicted that they should be an ancestral feature of this phylum. Indeed, phylogenetic analysis of the flagellar hook protein (FlgE) and flagellin (FliC, **Figure 3**) indicate a monophyletic origin of this trait in the Epsilonbacteraeota (Supplementary Figure S14). Flagella are known to be absent in several Epsilonbacteraeota, including *Nitratifractor salsuginis*, the genus *Sulfurovum*, and at least two *Campylobacter* species, *C. hominis* and *C. gracilis* (Vandamme et al., 1995; Lawson et al., 2001). The proposed Epsilonbacteraeota taxonomy groups the genera *Nitratifractor* and *Sulfurovum* into the novel family *Sulfurovaceae*, suggesting a single loss of flagella genes in the ancestor of this family. The presence of the chemotactic regulator Mcp and response regulating CheB and CheR within this family (**Figure 3**) provides additional evidence that the ancestor of the *Sulfurovaceae* possessed flagella. *Campylobacter hominis* and *C. gracilis* are sister species in the *Campylobacter* B clade (**Figure 2**) suggesting a single loss of motility occurred in their common ancestor for as yet, unclear reasons given that the rest of the genus has retained motility.

### Concluding Remarks

The rise of high-throughput DNA sequencing has fundamentally changed the landscape of microbial ecology and systematics. The 16S rRNA gene has led the way in providing an evolutionary backbone for microbial taxonomy over the past 30 years, but can now be complemented by whole genome methods which use multiple genetic markers to infer evolutionary relationships. More importantly, classifications are not static. As genomically described bacterial diversity expands, historic classifications require revision as new clades are discovered and tree topologies change. The class *Epsilonproteobacteria* and order *Desulfurellales* are an excellent case in point. Comparative analysis of these groups with a broad genomic representation of the bacterial domain indicates that they constitute a monophyletic unit not specifically related to the phylum Proteobacteria. We propose the reclassification of this group as the phylum Epsilonbacteraeota, together with a small number of subordinate changes described in this manuscript. Additional changes may be required in future as, e.g., *Campylobacter* and *Helicobacter* appear to be phylogenetically and functionally diverse for genera. Metabolic reconstruction of the Epsilonbacteraeota suggests, perhaps unsurprisingly, that the ancestor to this phylum was a chemolithoautotrophic thermophile related to Aquificae, from which several heterotrophic and mesophilic lineages have evolved.

### Proposed Taxonomy of Epsilonbacteraeota

#### Description of *Campylobacteria* class. nov.

*Campylobacteria* (Cam.py.lo.bac.te′ri.a. N.L. masc. n. *Campylobacter* type genus of the type order of the class; suff. *-ia*, ending to denote a class; N.L. neut. pl. n. *Campylobacteria* the class of the order *Campylobacterales*).

Description is the same as for the order *Campylobacterales* (Garrity et al., 2006b). Type order: *Campylobacterales*, phylum: Epsilonbacteraeota phyl. nov.

#### Description of *Arcobacteraceae* fam. nov.

*Arcobacteraceae* (Ar.co.bac.ter.a.ce′ae. N.L. masc. n. *Arcobacter* type genus of the family; suff. *-aceae*, ending to denote a family; N.L. fem. pl. n. *Arcobacteraceae* the family of the genus *Arcobacter*).

Description is identical to that given by Vandamme et al. (1991), with the acknowledgment that some species are capable of autotrophic carbon dioxide fixation via the reverse tricarboxylic acid cycle (Hügler et al., 2005). Type genus: *Arcobacter*, order: *Campylobacterales*, class: *Campylobacteria*, class. nov., phylum: Epsilonbacteraeota phyl. nov.

#### Emended Description of the Family *Campylobacteraceae* Vandamme and De Ley (1991)

Description is drawn from that of Vandamme and De Ley (1991), with the removal of the genera *Arcobacter* and *Sulfurospirillum* due to a lack of robust monophyly with the type genus. Type genus: *Campylobacter*, order: *Campylobacterales*, class: *Campylobacteria* class. nov., phylum: Epsilonbacteraeota phyl. nov.

#### Emended Description of the Family *Helicobacteraceae* Garrity et al. (2006a)

The description is the same as given by Garrity et al. (2006a) for the type genus with the following changes. Currently includes only the genera *Helicobacter* (type genus) and *Wolinella*. The genera *Sulfurimonas*, *Sulfuricurvum*, *Sulfurovum*, and *Thiovulum* are removed owing to a lack of monophyly with the type genus. Type genus: *Helicobacter*, order: *Campylobacterales*, class: *Campylobacteria* class. nov., phylum: Epsilonbacteraeota phyl. nov.

#### Description of *Nitratiruptoraceae* fam. nov.

*Nitratiruptoraceae* (Ni.tra.ti.rup.to.ra.ce′ae. N.L. masc. n. *Nitratiruptor* type genus of the family; suff. *-aceae*, ending to denote a family; N.L. fem. pl. n. *Nitratiruptoraceae* the family of the genus *Nitratiruptor*).

Described on the basis of divergent nature with all other members of *Campylobacterales* using both protein marker, as well as 16S and 23S rRNA gene sequence analysis. Description is the same as given by Nakagawa et al. (2005). Type genus: *Nitratiruptor*, order: *Campylobacterales*, class: *Campylobacteria* class. nov., phylum: Epsilonbacteraeota phyl. nov.

#### Description of *Sulfurospirillaceae* fam. nov.

*Sulfurospirillaceae* (Sul.fu.ro.spir.il.la.ce′ae. N.L. neut. n. *Sulfurospirillum* type genus of the family; suff. *-aceae*, ending to denote a family; N.L. fem. pl. n. S*ulfurospirillaceae* the family of the genus *Sulfurospirillum*).

The description of the family is drawn from the description of the type genus from Schumacher et al. (1992); cells are Gram-negative, motile, microaerobic, and mesophilic. Oxidize hydrogen or formate to reduce a range of electron acceptors. Type genus: *Sulfurospirillum*, order: *Campylobacterales*, class: *Campylobacteria* class. nov., phylum: Epsilonbacteraeota phyl. nov.

#### Description of *Sulfurovaceae* fam. nov.

*Sulfurovaceae* (Sul.fur.o.va.ce′ae. N.L. neut. n. *Sulfurovum* type genus of the family; suff. *-aceae*, ending to denote a family; N.L. fem. pl. n. *Sulfurovaceae* the family of the genus *Sulfurovum*).

The family is defined based on phylogenetic analysis of protein markers (concatenated and single) and the 16S ribosomal gene sequence. Includes the genera *Sulfurovum* (type genus) and *Nitratifractor*. Cells are Gram-negative, non-motile, and mesophilic. Growth occurs in the microaerobic range. Type genus: *Sulfurovum*, order: *Campylobacterales*, class: *Campylobacteria* class. nov., phylum: Epsilonbacteraeota phyl. nov.

#### Description of *Thiovulaceae* fam. nov.

*Thiovulaceae* (Thio.vu.la.ce’ae. N.L. neut. dim. n. *Thiovulum* type genus of the family; suff. *-aceae*, ending to denote a family; N.L. fem. pl. n. *Thiovulaceae* the family of the genus *Thiovulum*).

The family is defined based on phylogenetic analysis of concatenated protein marker and ribosomal gene sequences. The family currently includes the genera *Thiovulum* (type genus), *Sulfurimonas*, and *Sulfuricurvum*. Cells are Gram-negative, motile, microaerobic, and mesophilic. Growth occurs in the mesophilic range. Type genus: *Thiovulum*, order: *Campylobacterales*, class: *Campylobacteria*class. nov., phylum: Epsilonbacteraeota phyl. nov.

#### Description of *Desulfurellia* class. nov.

*Desulfurellia* (De.sul.fu.rel′li.a. N.L. fem. n. *Desulfurella* type genus of the type order of the class; suff. *-ia*, ending to denote a class; N.L. neut. pl. n. *Desulfurellia* the class of the order *Desulfurellales*).

Description is the same as for the order *Desulfurellales* (Kuever et al., 2006b). Type order: *Desulfurellales*, phylum: Epsilonbacteraeota phyl. nov.

#### Emended Description of the Order *Desulfurellales* Kuever et al. (2006b)

The order is as previously described (Kuever et al., 2006b) but is transferred from the class *Deltaproteobacteria* to *Desulfurellia* class. nov. The order consists of the families *Desulfurellaceae* and *Hippeaceae* fam. nov. Cells are Gram-negative, thermophilic, and motile. Type family: *Desulfurellaceae*, class: *Desulfurellia* class. nov., phylum: Epsilonbacteraeota phyl. nov.

#### Emended Description of the Family *Desulfurellaceae* Kuever et al. (2006a)

Description is as previously (Kuever et al., 2006a), with the exception that the genus *Hippea* is moved to the family *Hippeaceae* fam. nov.. Cells are Gram-negative, obligately anaerobic, and motile. Uses short-chain fatty acids including acetate, fumarate, malate, as well as long-chain saturated fatty acids as growth substrates. Type species *D. acetivorans* is capable of dissimilatory sulfur reduction (Bonch-Osmolovskaya et al., 1990). Type genus: *Desulfurella*, order: *Desulfurellales*, class: *Desulfurellia* class. nov., phylum: Epsilonbacteraeota phyl. nov.

#### Description of *Hippeaceae* fam. nov.

*Hippeaceae* (Hippe.a.ce′ae. N.L. fem. n. *Hippea* type genus of the family; suff. *-aceae*, ending to denote a family; N.L. fem. pl. n. *Hippeaceae* the family of the genus *Hippea*).

The family is defined based on phylogenetic analysis of concatenated marker proteins, and is separated from the *Desulfurellaceae* due to deep branching distance. Cells are Gram-negative, obligately anaerobic, thermophilic, and motile. Capable of heterotrophic growth using acetate and various other organic substrates by species. Type genus: *Hippea*, order: *Desulfurellales*, class: *Desulfurellia* class. nov., phylum: Epsilonbacteraeota phyl. nov.

#### Description of Epsilonbacteraeota phyl. nov.

Epsilonbacteraeota (Ep.si.lon.bac.ter.ae.o′ta. Gr. n. *epsilon*, name of the fifth letter of Greek alphabet; Gr. n. *baktêria*, staff, cane; suff. *-aeota*, proposed ending to denote a phylum; N.L. neut. pl. n. Epsilonbacteraeota the phylum of bacteria sharing a common ancestry and possessing ribosomal gene sequence and protein marker similarity to those of the members of the classes *Campylobacteria* class. nov. and *Desulfurellia* class. nov)

The phylum is described based upon concatenated protein marker and ribosomal gene sequence phylogeny. Owing to the diverse ecological range of members of this phylum there is little unifying physiology. All members are Gram-negative, and motility as well as the reduction of nitrate and polysulfide are ancestral and common traits. The phylum Epsilonbacteraeota currently comprises two classes: *Campylobacteria* class. nov. and *Desulfurellia* class. nov.; and three orders: *Campylobacterales*, *Nautiliales*, and *Desulfurellales*. The type class of the phylum is *Campylobacteria* class. nov.

## Author Contributions

DW, DP, and PH performed the bioinformatic analysis, interpretation, and proposed taxonomic changes. KT, MK, SS, and TW sequenced additional genomes for analysis. BC, IV, JS, and MK proposed avenues for functional analysis and assisted with interpretation of data. DW and PH wrote the draft manuscript and all authors contributed to, and approved, the final manuscript.

## Conflict of Interest Statement

The authors declare that the research was conducted in the absence of any commercial or financial relationships that could be construed as a potential conflict of interest.

## Footnotes

1 https://gold.jgi.doe.gov/

2 http://gtdb.ecogenomic.org/downloads

3 https://github.com/wwood/finishm

4 https://github.com/dparks1134/RefineM

5 https://github.com/geronimp/graftM

6 https://github.com/dparks1134/CompareM

7 https://github.com/Ecogenomics/mingle

8 gtdb.ecogenomic.org
