# Addendum: Comparative Genomic Analysis of the Class *Epsilonproteobacteria* and Proposed Reclassification to Epsilonbacteraeota (phyl. nov.)

**DOI:** 10.3389/fmicb.2018.00772

**Published:** 2018-04-18

**Authors:** David W. Waite, Inka Vanwonterghem, Christian Rinke, Donovan H. Parks, Ying Zhang, Ken Takai, Stefan M. Sievert, Jörg Simon, Barbara J. Campbell, Thomas E. Hanson, Tanja Woyke, Martin G. Klotz, Philip Hugenholtz

**Affiliations:** ^1^Australian Centre for Ecogenomics, School of Chemistry and Molecular Biosciences, The University of Queensland, St Lucia, QLD, Australia; ^2^Department of Cell and Molecular Biology, College of the Environment and Life Sciences, University of Rhode Island, Kingston, RI, United States; ^3^Department of Subsurface Geobiological Analysis and Research (D-SUGAR), Japan Agency for Marine-Earth Science and Technology, Yokosuka, Japan; ^4^Department of Biology, Woods Hole Oceanographic Institution, Woods Hole, MA, United States; ^5^Microbial Energy Conversion and Biotechnology, Department of Biology, Technische Universität Darmstadt, Darmstadt, Germany; ^6^Department of Biological Sciences, Life Science Facility, Clemson University, Clemson, SC, United States; ^7^School of Marine Science and Policy, College of Earth, Ocean, and Environment, University of Delaware, Newark, DE, United States; ^8^Delaware Biotechnology Institute, University of Delaware, Newark, DE, United States; ^9^Department of Energy, Joint Genome Institute, Walnut Creek, CA, United States; ^10^School of Molecular Biosciences, College of Veterinary Medicine, Washington State University, Pullman, WA, United States; ^11^State Key Laboratory of Marine Environmental Science, Institute of Marine Microbes and Ecospheres, College of Ocean and Earth Sciences, Xiamen University, Xiamen, China

**Keywords:** *Epsilonproteobacteria*, taxonomy, classification, genome, phylogenomics, Epsilonbacteraeota, Epsilonbacterota, evolution

In our original publication, we proposed the phylum name Epsilonbacteraeota according to a proposal to modify Rule 8 of the International Code of Nomenclature of Prokaryotes, under which the suffix –*aeota* would be used to denote prokaryotic phyla (Oren et al., 2015). An addendum to this proposal was recently made whereby the shorter suffix –*ota*, instead of –*aeota*, be added to the stem of the name of one of the contained classes (Whitman et al., 2018). In accordance with this amendment, and the requirement that a class name is derived from a type genus (Oren et al., 2015), we propose to replace the name Epsilonbacteraeota with Campylobacterota. This change does not affect subordinate ranks of the phylum.

**Campylobacterota** (Cam.py.lo.bac.ter.o′ta. N.L. neut. n. *Campylobacter* type genus of the type order of the type class of the phylum; suff. *-ota*, proposed ending to denote a phylum; N.L. neut. pl. n. Campylobacterota the phylum of the class *Campylobacteria*).

## Conflict of interest statement

The authors declare that the research was conducted in the absence of any commercial or financial relationships that could be construed as a potential conflict of interest.
