# Supplementary material for: Comparative Genomic Analysis of the Class Epsilonproteobacteria and Proposed Reclassification to Epsilonbacteraeota (phyl. nov.)
Source: Front Microbiol. 2017 Apr 24;8:682. doi: 10.3389/fmicb.2017.00682 (PMC5401914; doi:10.3389/fmicb.2017.00682)
Supplement: Supplementary file 2 [file Data_Sheet_1.DOCX]

Supplementary Material

Comparative genomic analysis of the class *Epsilonproteobacteria* and proposed reclassification to Epsilonbacteraeota (*phyl. nov.*)

David W. Waite, Inka Vanwonterghem, Christian Rinke, Donovan H. Parks, Ying Zhang, Ken Takai, Stefan M. Sievert, Jörg Simon, Barbara J. Campbell, Thomas E. Hanson, Tanja Woyke, Martin G. Klotz, Philip Hugenholtz*

*** Correspondence:** Philip Hugenholtz: p.hugenholtz@uq.edu.au


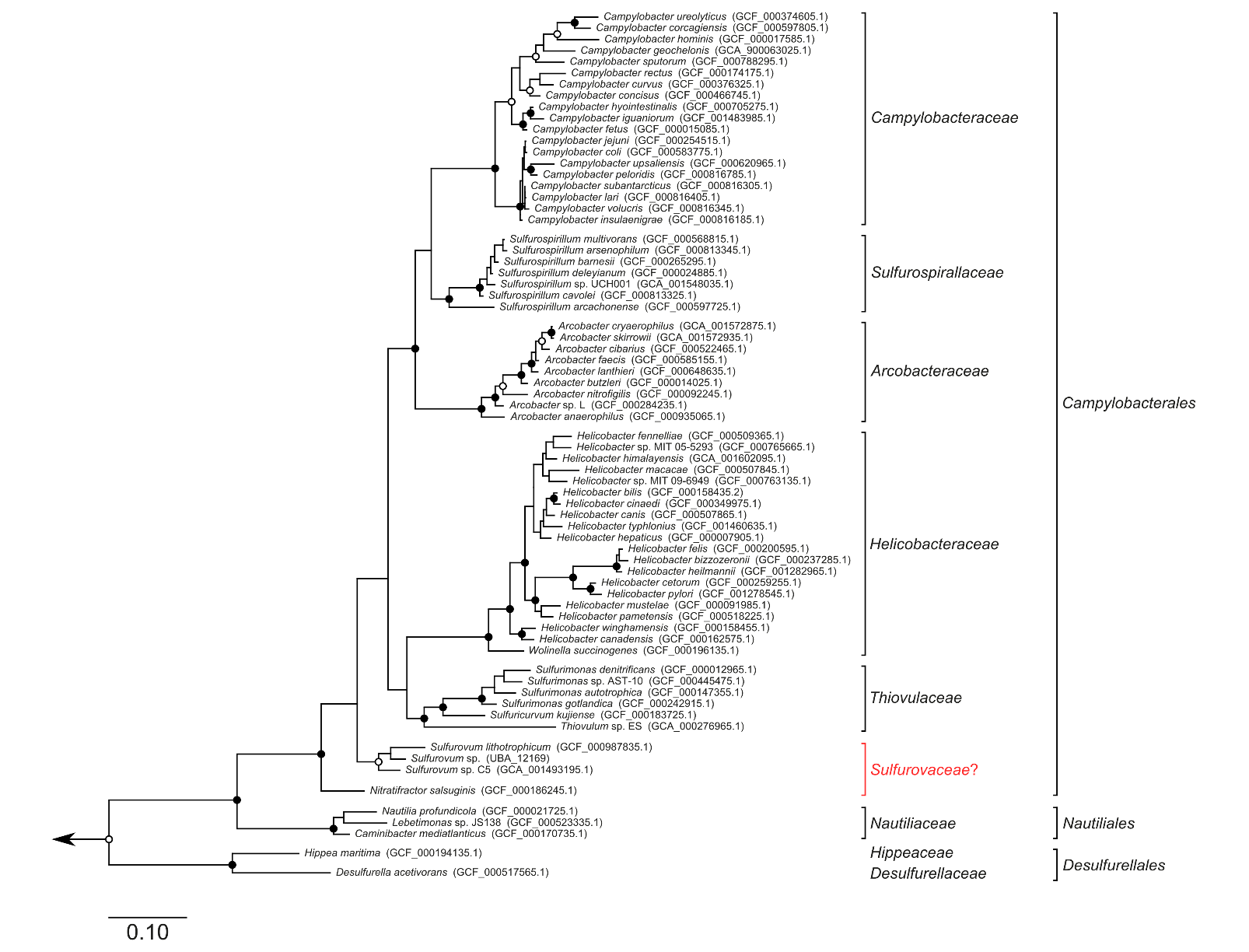


**Supplemental Figure S1:** Maximum likelihood phylogenetic inference of concatenated 16S and 23S rRNA sequences. Tree robustness was assessed with 1,000 bootstrap iterations. Symbols denote support >90% (solid) or >75% (hollow). Under this analysis the proposed family *Sulfurovaceae* (red) is not monophyletic.

**
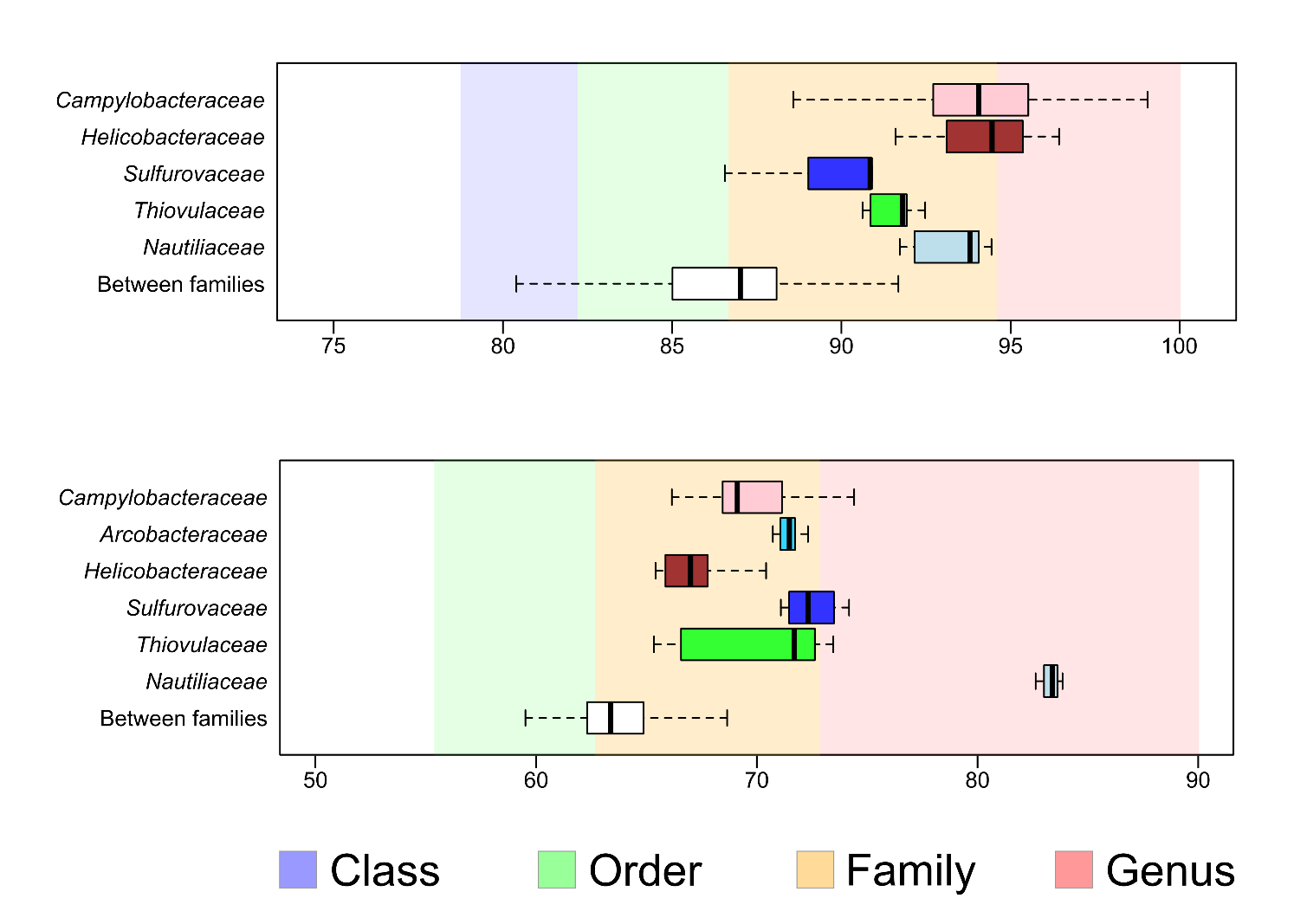
**

**Supplemental Figure S2:** Comparison of Epsilonbacteraeota to previously described family boundaries. As comparisons are only made between-genera some families are omitted. *Campylobacteraceae* similarities were calculated using the proposed genus groupings proposed in **Figure 2**. Top: 16S rRNA sequence similarity compared to the boundaries proposed by Yarza et al. (2014). Bottom: Amino acid identities compared to boundaries extrapolated from Konstantinidis and Tiedje (2005). Note that the values in this study consisted of overlapping ranges, which have not been drawn for clarity. Boundaries are instead plotted as the midpoint between boundary overlap.


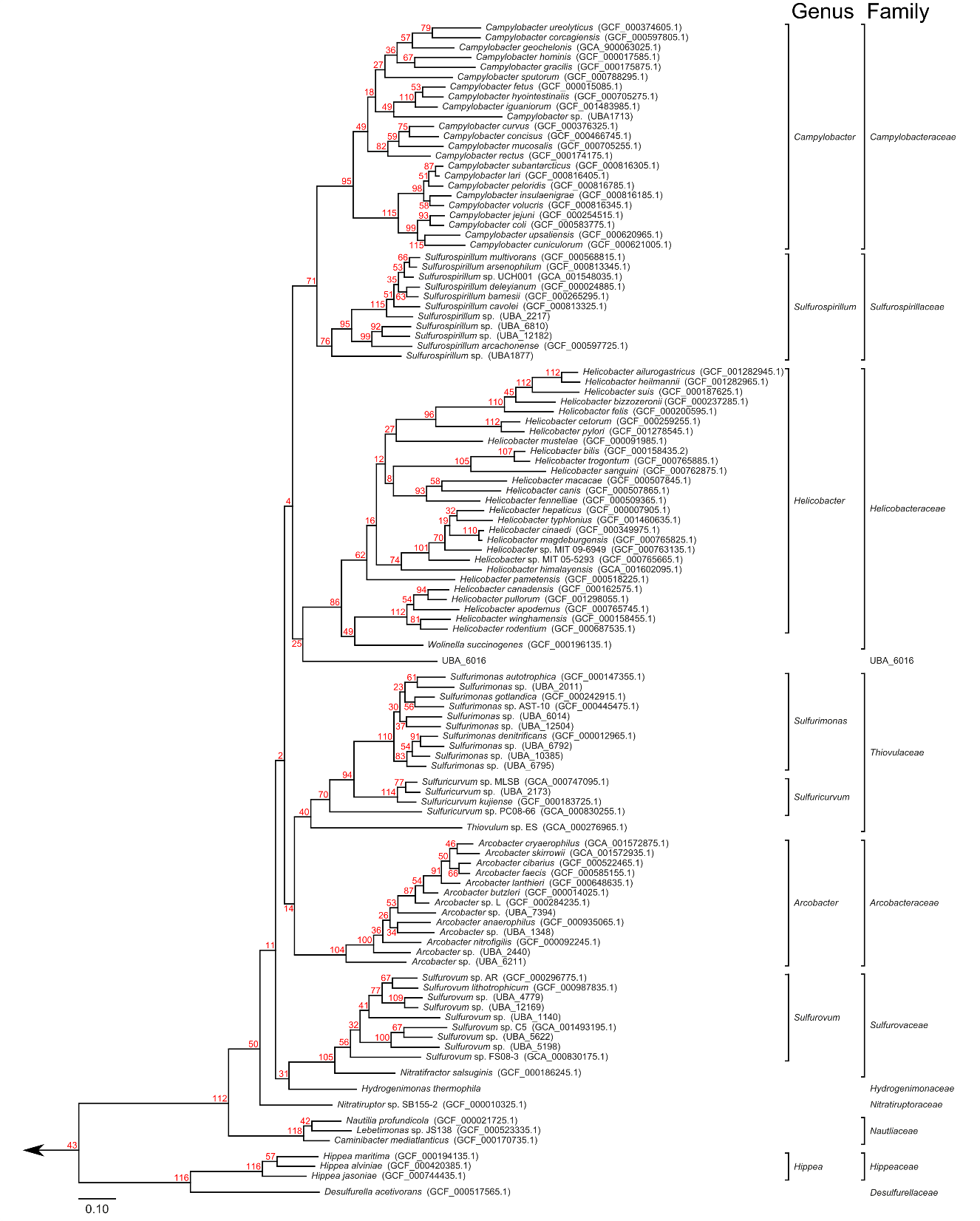


**Supplemental Figure S3:** Consensus topology of single protein marker phylogenetic analyses. Individual tree topologies were calculated for each individual protein marker and assessed for consistency with the concatenated marker tree topology (displayed). For each junction in the concatenated tree, the child leaf nodes (genomes) were tested for monophyly in each single marker tree. Results are reported for each junction in red. Note that as not every protein is present in every genome, counts are not necessarily a fraction of 120 total tree topologies.


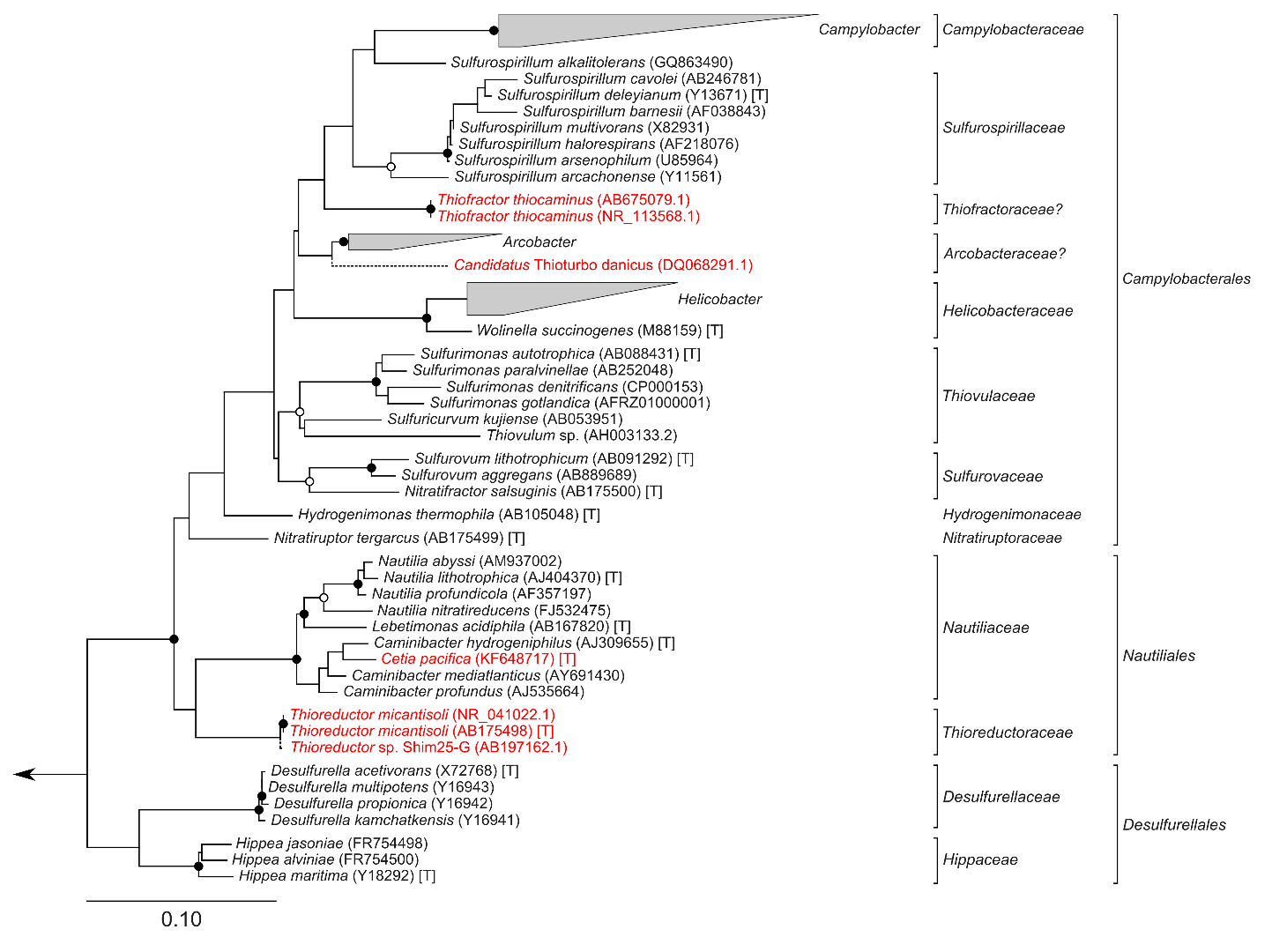


**Supplemental Figure S4:** Maximum likelihood phylogenetic inference of 16S rRNA sequences. Reference sequences (black) were obtained from the SILVA Living Tree Project (release 123), with the exception of *Thiovulum* sp. which was obtained from NCBI GenBank. Putative Epsilonbacteraeota for which genomes are not available (red) were tested for their placement within this data set. Tree robustness was assessed by 1,000 bootstrap iterations with support denoted by solid (>90%) or hollow (>75%) junctions. Only a partial sequence was available for *Candidatus* Thioturbo danicus, and this sequence was inserted into the main tree using the Parsimony Insertion tool in ARB following tree construction. [T]: denotes type strain. SILVA LTP or GenBank accessions are provided in brackets.


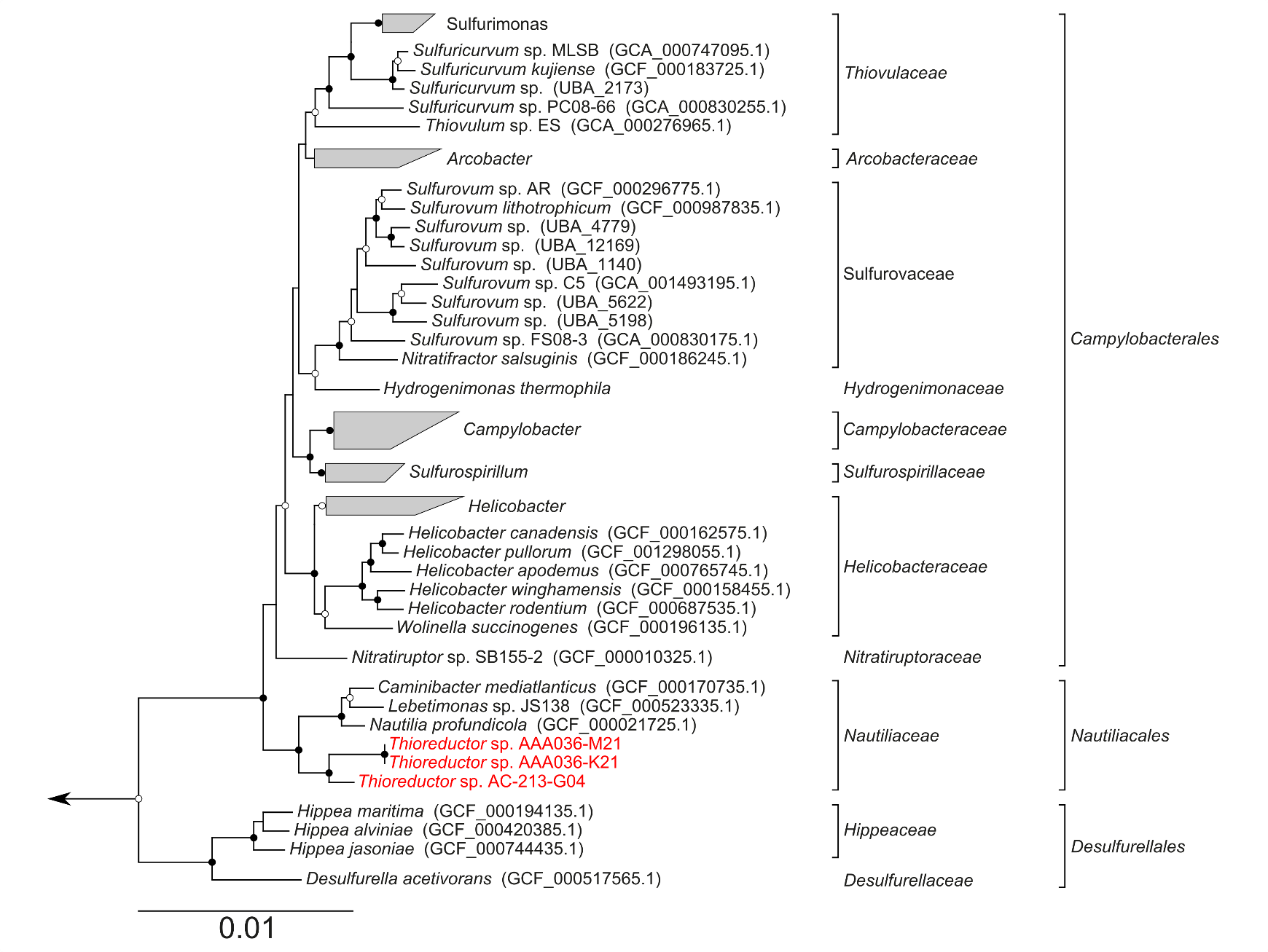


**Supplemental Figure S5:** Maximum likelihood phylogenetic inference of concatenated protein markers present in *Thioreductor* genomes. Concatenated alignment of 14 protein markers common to all *Thioreductor* partial genomes. Tree robustness was assessed with 100 bootstrap iterations with junctions denoting >90% support (solid) or >75% (hollow). A number of *Campylobacterales* genera are collapsed for clarity.


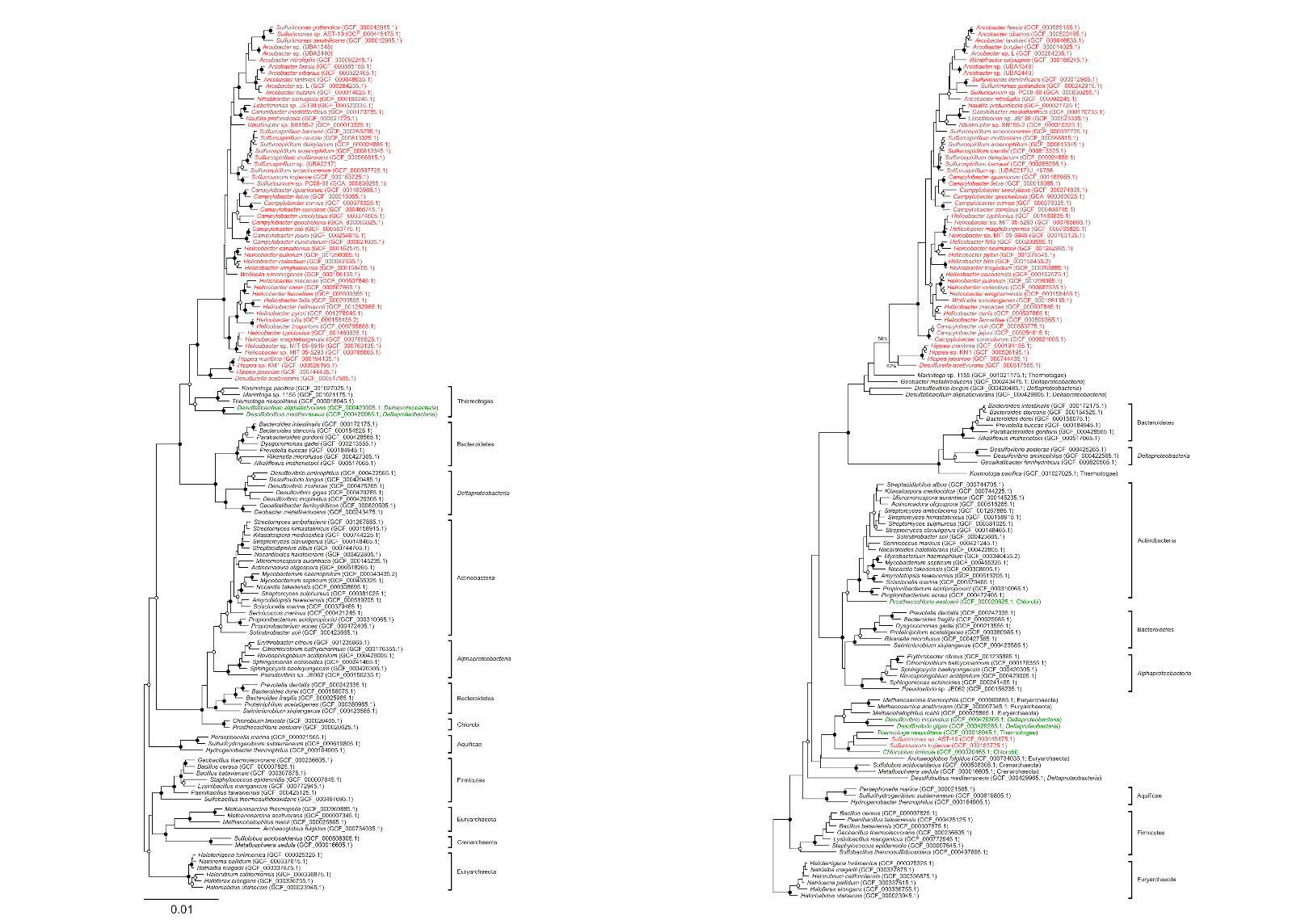


**Supplemental Figure S6:** Phylogenetic inference of 2-oxoglutarate oxidoreductase subunits from 128 representative genomes. Left: Alpha subunit (KorA), consisting of 530 amino acid positions following alignment trimming. Right: Beta subunit (KorB), consisting of 276 amino acid positions following alignment trimming. Tree robustness was assessed using 100 bootstrap iterations with junctions denoting >90% support (solid) or >70% (hollow). Note that the support of Epsilonbacteraeota in KorB is expressed numerically as it does not fit this criteria. Epsilonbacteraeota sequences are marked in red, and putative lateral gene transfers in green.


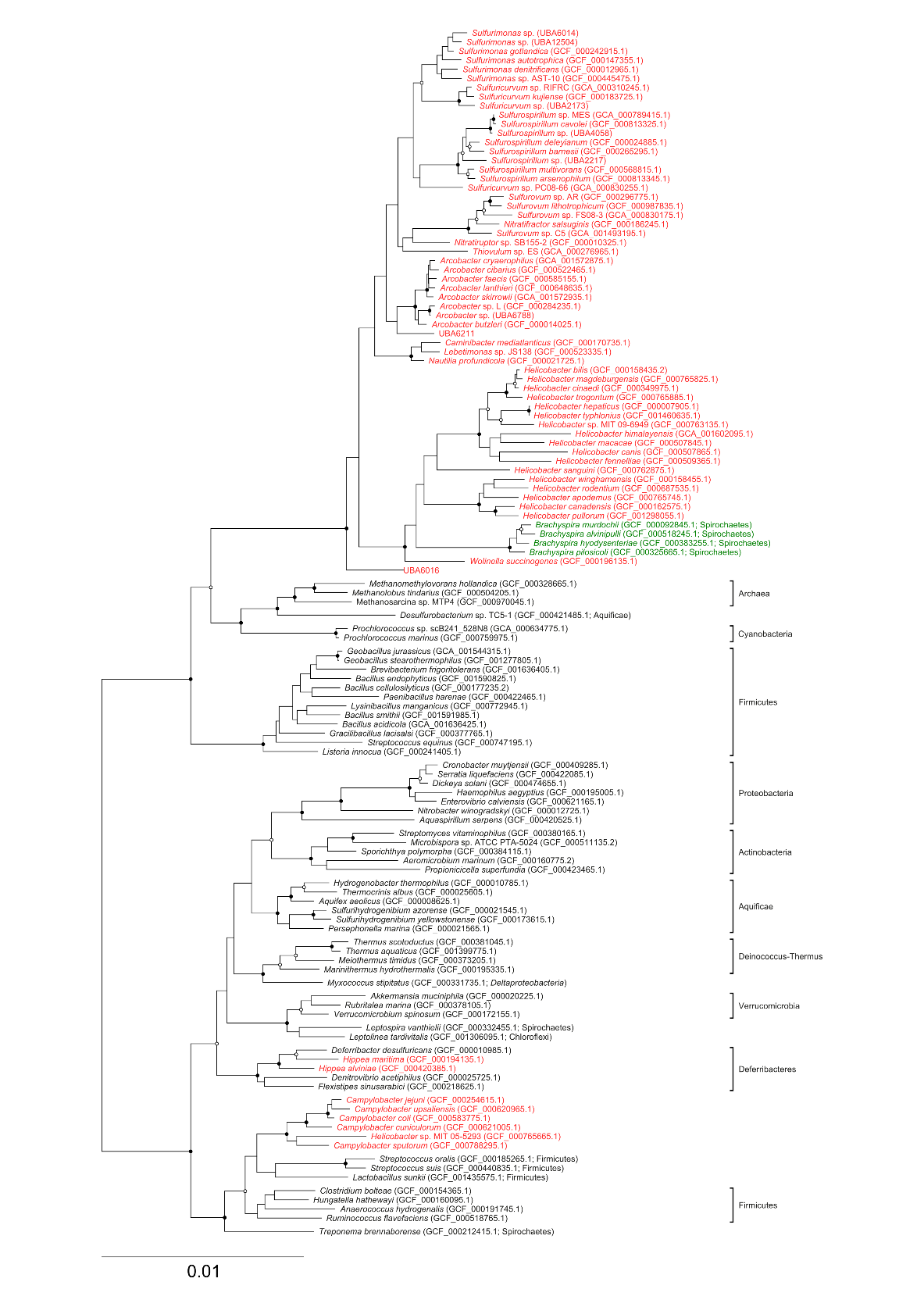


**Supplemental Figure S7:** Phylogenetic inference of methylene-tetrahydrofolate reductase (MetF) from 125 representative genomes. Alignment consisted of 179 amino acid positions following trimming. Tree robustness was assessed using 100 bootstrap iterations with junctions denoting >90% support (solid) or >70% (hollow). Epsilonbacteraeota sequences are marked in red, and putative lateral gene transfers in green. Note that the *Campylobacterales* are not monophyletic, and that a lateral transfer of MetF from host-associated Firmicutes species is predicted to have caused this result.


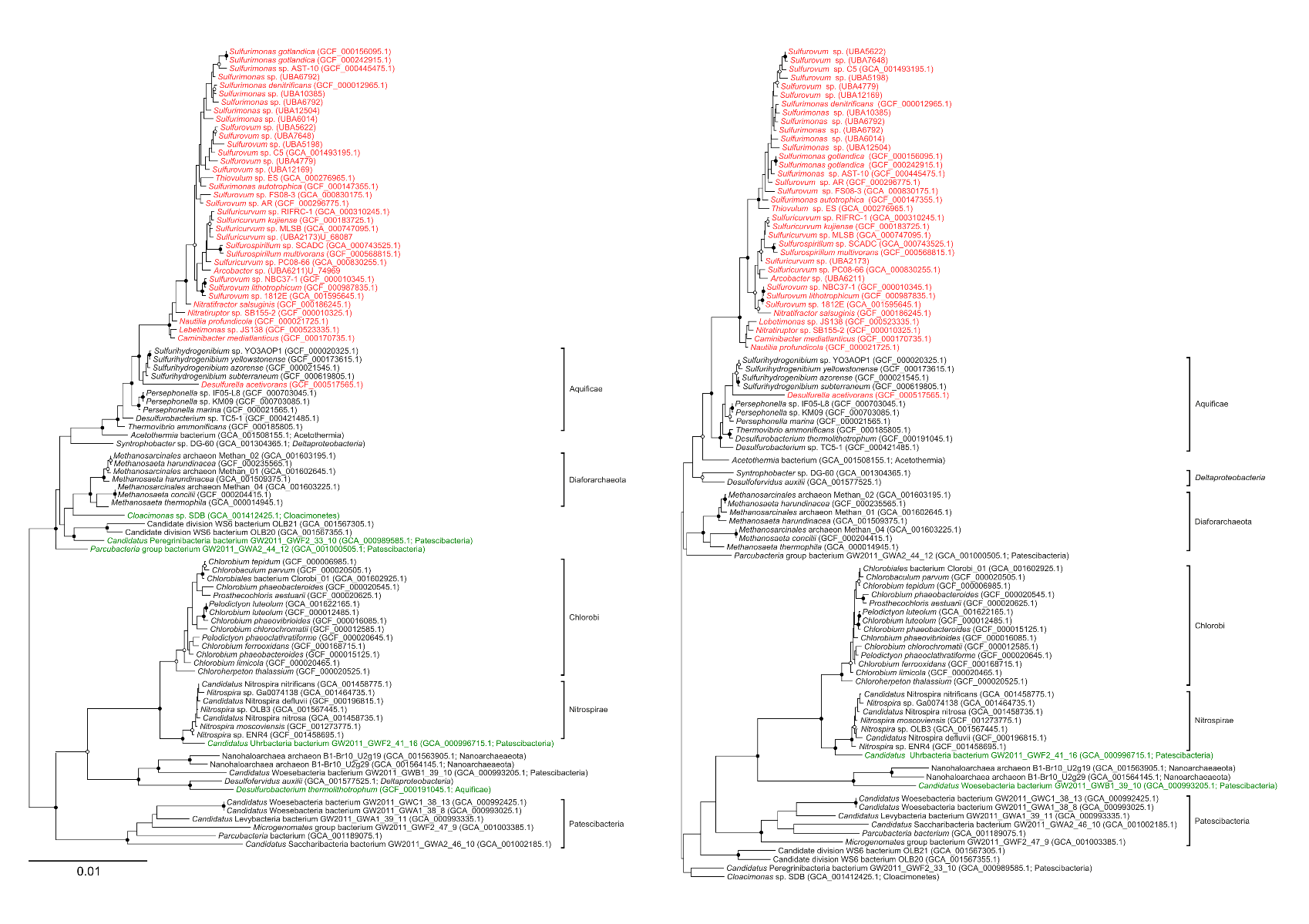


**Supplemental Figure S8:** Phylogenetic inference of ATP citrate lyase subunits from 95 representative genomes. Left: Alpha subunit (AclA), consisting of 271 amino acid positions following alignment trimming. Right: Beta subunit (AclB), consisting of 368 amino acid positions following alignment trimming. Tree robustness was assessed using 100 bootstrap iterations with junctions denoting >90% support (solid) or >70% (hollow). Epsilonbacteraeota sequences are marked in red, and putative lateral gene transfers in green.


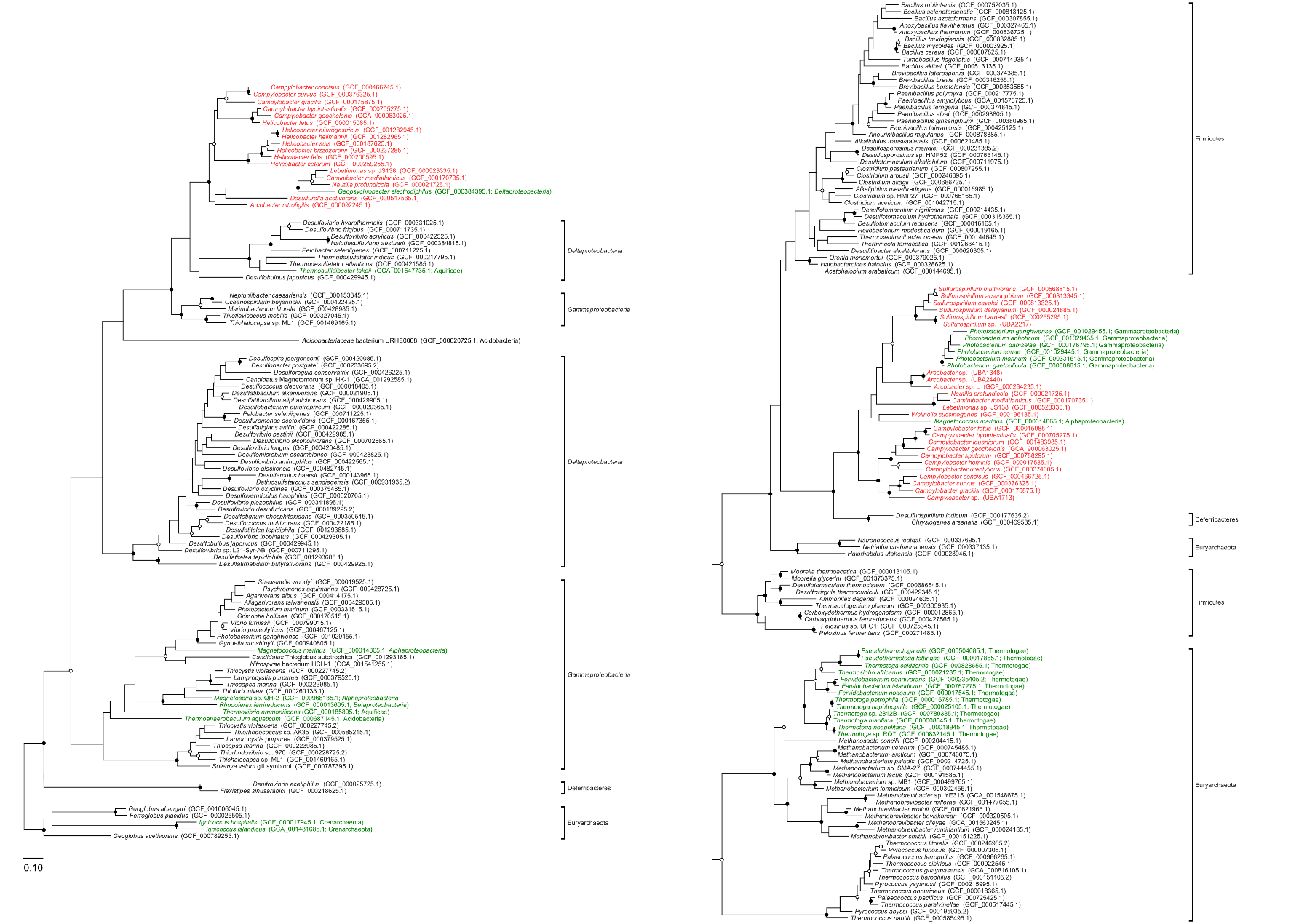


**Supplemental Figure S9:** Phylogenetic inference of reverse-HURM components. Left: Hydroxyamine oxidoredutase (HaoA) from 103 representative genomes, consisting of 329 amino acid positions following alignment trimming. Right: Hydroxylamine reductase (Har), consisting of 425 amino acids following alignment trimming. Tree robustness was assessed using 100 bootstrap iterations with junctions denoting >90% support (solid) or >70% (hollow). Epsilonbacteraeota sequences are marked in red, and putative lateral gene transfers in green.


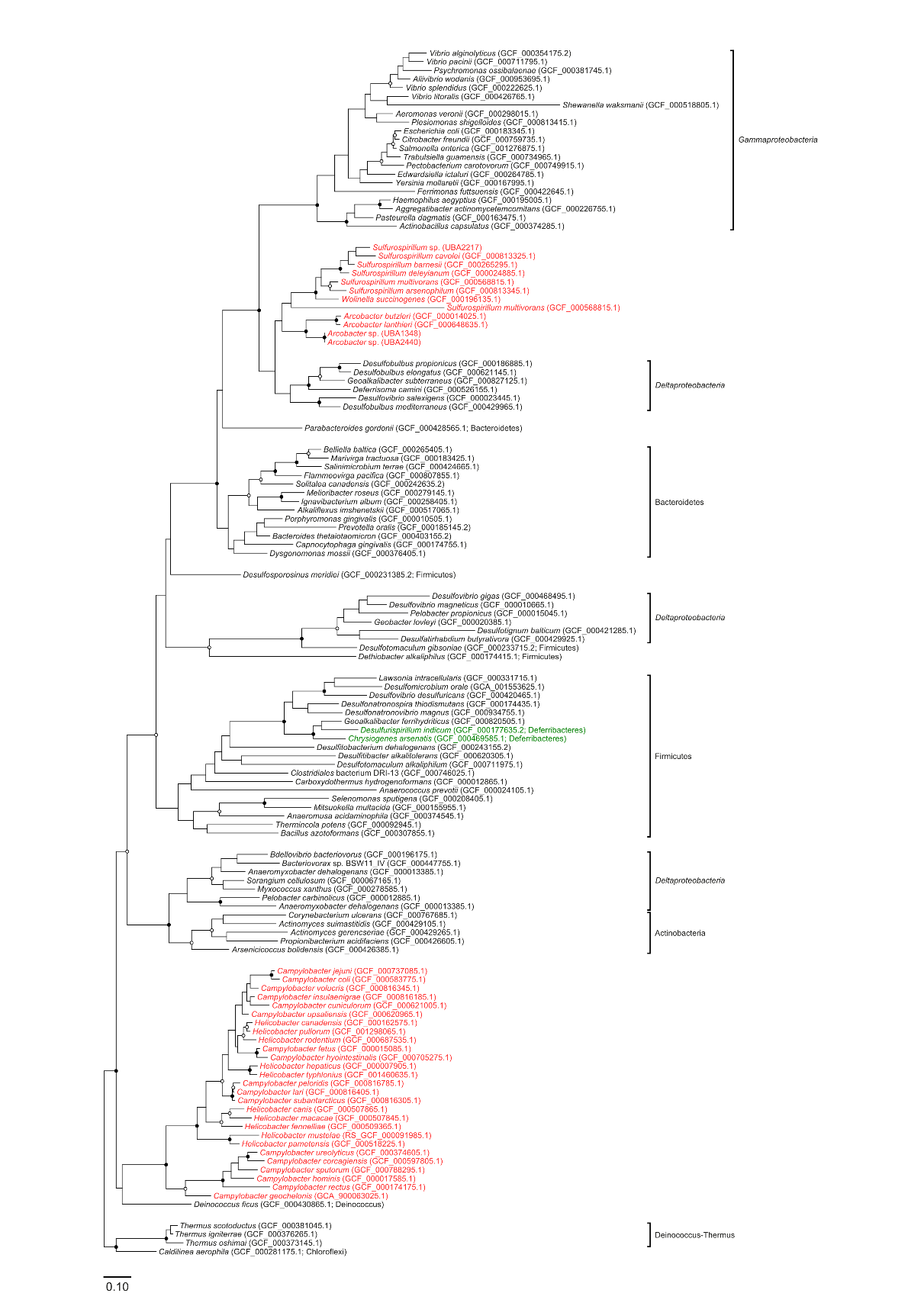


**Supplemental Figure S10:** Phylogenetic inference of pentaheme cytochrome *c* nitrite reductase from 127 representative genomes. NrfA subunit, consisting of 238 amino acid positions following alignment trimming. Tree robustness was assessed using 100 bootstrap iterations with junctions denoting >90% support (solid) or >70% (hollow). Epsilonbacteraeota sequences are marked in red, and putative lateral gene transfers in green.


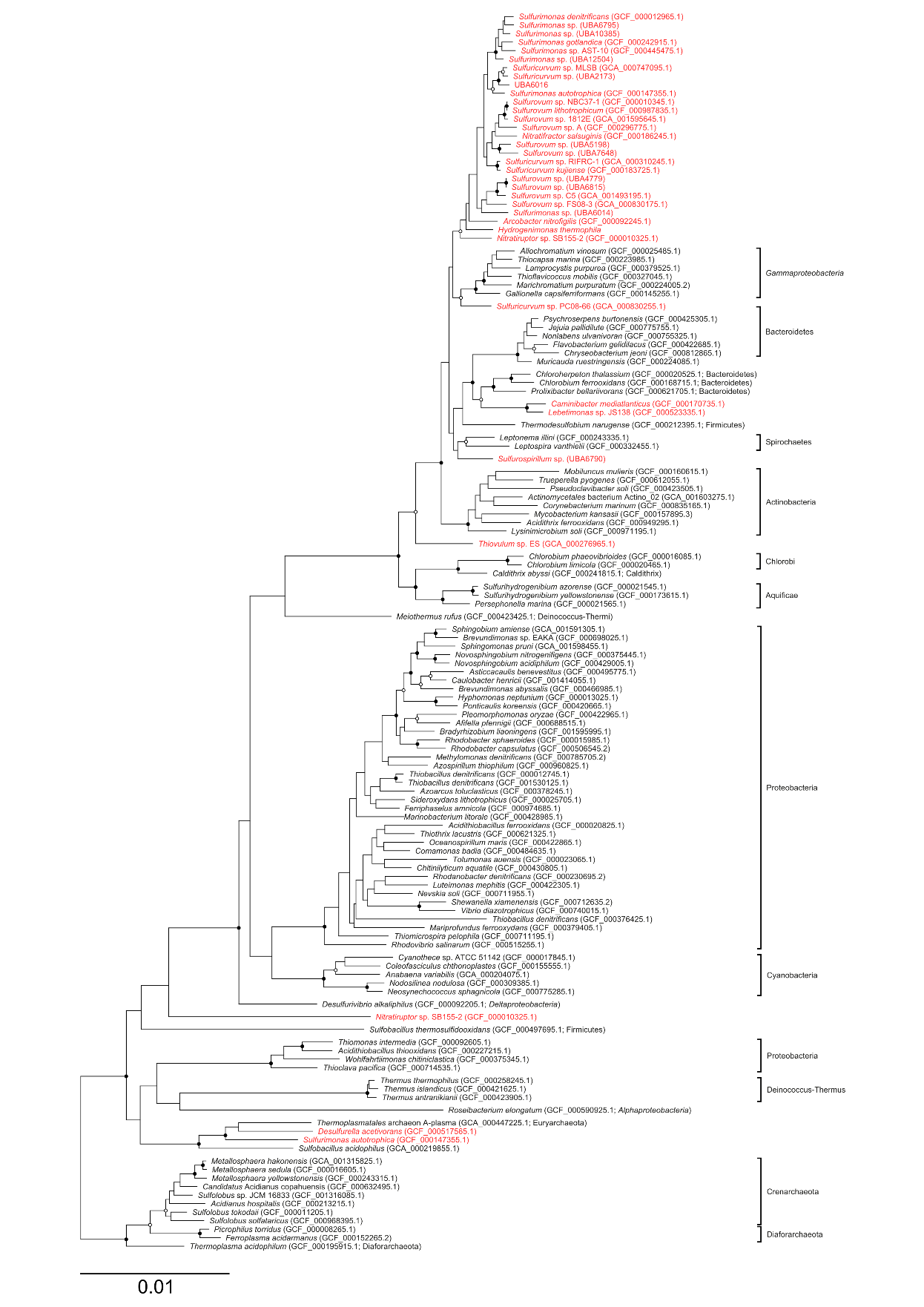


**Supplemental Figure S11:** Phylogenetic inference of sulfide:quinone oxidoreductase from 135 representative genomes. Alignment consisted of 142 amino acid positions following trimming. Tree robustness was assessed using 100 bootstrap iterations with junctions denoting >90% support (solid) or >70% (hollow). Epsilonbacteraeota sequences are marked in red, and putative lateral gene transfers in green.


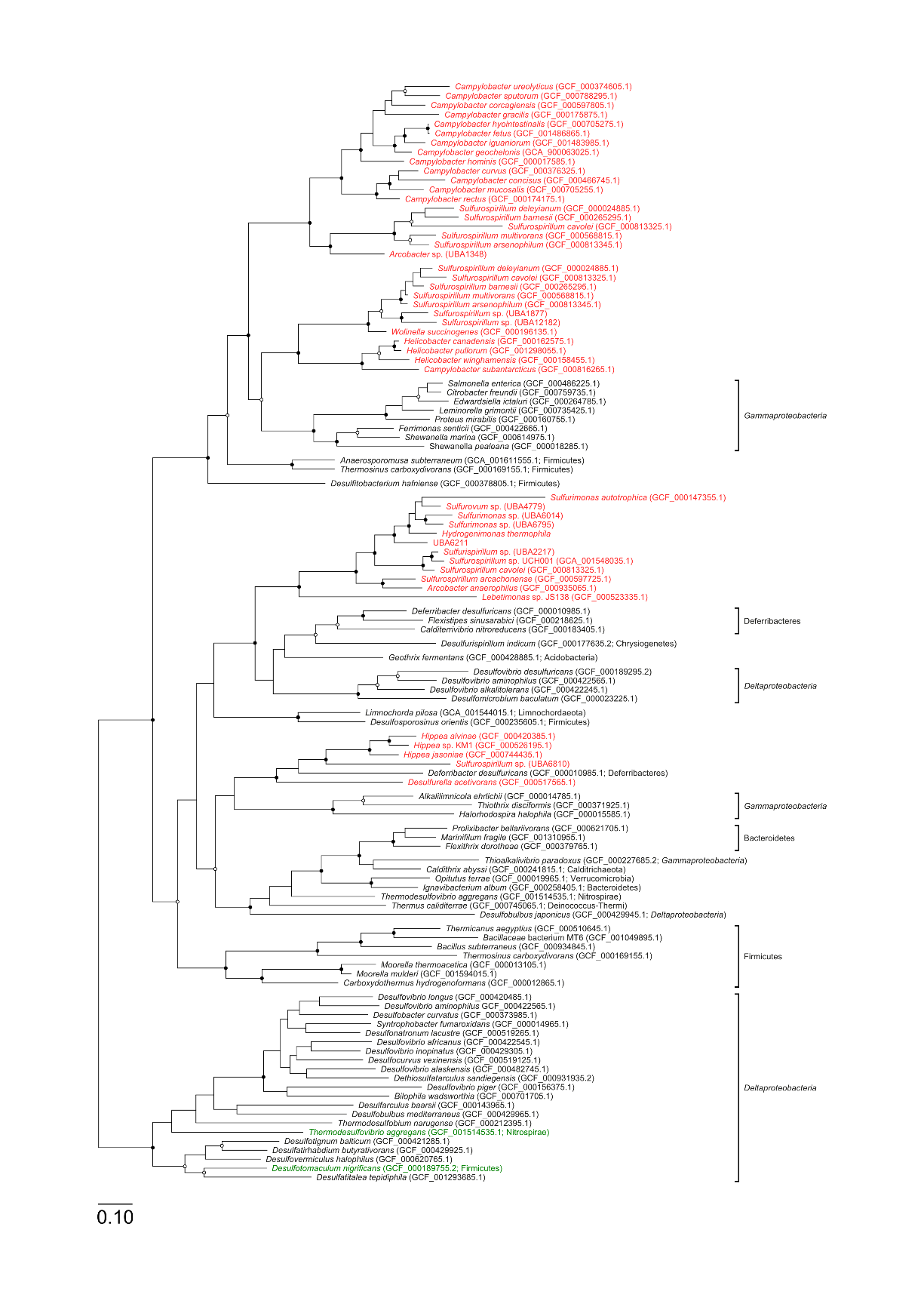


**Supplemental Figure S12:** Phylogenetic inference of polysulfide reductase chain A from 112 representative genomes. Alignment consisted of 441 amino acid positions following trimming. Tree robustness was assessed using 100 bootstrap iterations with junctions denoting >90% support (solid) or >70% (hollow). Epsilonbacteraeota sequences are marked in red, and putative lateral gene transfers in green.


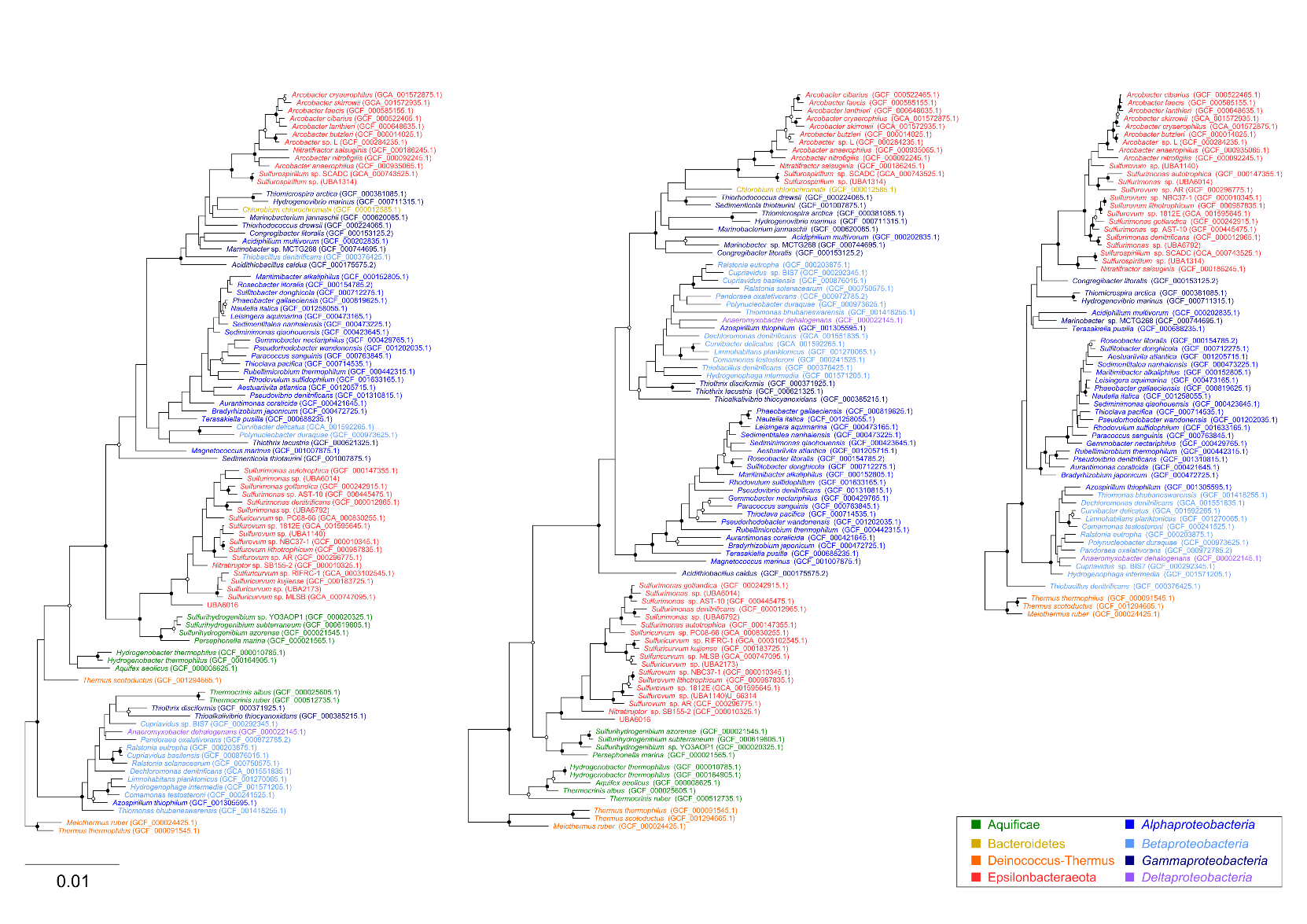


**Supplemental Figure S13:** Phylogenetic inference of Sox proteins. Left: SoxA from 90 representative genomes, consisting of 167 amino acid positions following alignment trimming. Middle: SoxB, consisting of 506 amino acid positions following trimming. Right: 403 amino acid alignment of the SoxC protein, from a subset of 63 genomes. Tree robustness was assessed using 100 bootstrap iterations with junctions denoting >90% support (solid) or >70% (hollow).


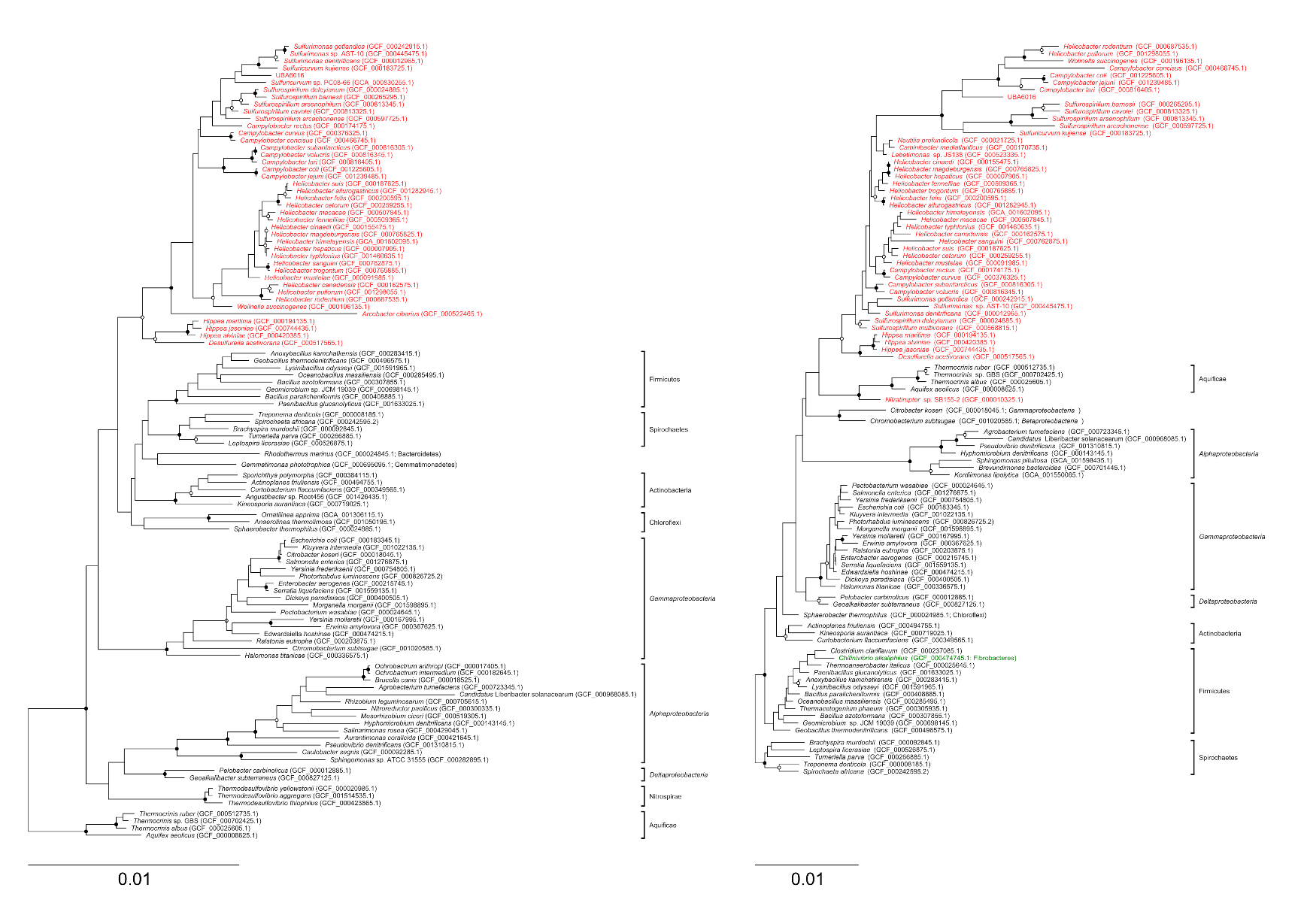


**Supplemental Figure S14:** Phylogenetic inference of flagella structural proteins. Left: Flagellar hook protein (FlgE) from 105 representative genomes, consisting of 213 amino acid positions following alignment trimming. Right: Flagellin protein from 96 genomes and 148 amino acid alignment positions. Tree robustness was assessed using 100 bootstrap iterations with junctions denoting >90% support (solid) or >70% (hollow). Epsilonbacteraeota sequences are marked in red, and putative lateral gene transfers in green.
